# Variation in genomic vulnerability to climate change across temperate populations of eelgrass (*Zostera marina*)

**DOI:** 10.1111/eva.13671

**Published:** 2024-04-21

**Authors:** Nicholas W. Jeffery, Benedikte Vercaemer, Ryan R. E. Stanley, Tony Kess, France Dufresne, Fanny Noisette, Mary I. O'Connor, Melisa C. Wong

**Affiliations:** ^1^ Fisheries and Oceans Canada Bedford Institute of Oceanography Dartmouth Nova Scotia Canada; ^2^ Fisheries and Oceans Canada, Northwest Atlantic Fisheries Centre St. John's Newfoundland and Labrador Canada; ^3^ Département de Biologie Université du Québec à Rimouski Rimouski Quebec Canada; ^4^ Institut des Sciences de la mer, Université du Québec à Rimouski Rimouski Quebec Canada; ^5^ Department of Zoology and Biodiversity Research Centre University of British Columbia Vancouver British Columbia Canada

**Keywords:** Atlantic Ocean, conservation genomics, genome resequencing, poolseq, seagrass

## Abstract

A global decline in seagrass populations has led to renewed calls for their conservation as important providers of biogenic and foraging habitat, shoreline stabilization and carbon storage. Eelgrass (*Zostera marina*) occupies the largest geographic range among seagrass species spanning a commensurately broad spectrum of environmental conditions. In Canada, eelgrass is managed as a single phylogroup despite occurring across three oceans and a range of ocean temperatures and salinity gradients. Previous research has focused on applying relatively few markers to reveal population structure of eelgrass, whereas a whole‐genome approach is warranted to investigate cryptic structure among populations inhabiting different ocean basins and localized environmental conditions. We used a pooled whole‐genome re‐sequencing approach to characterize population structure, gene flow and environmental associations of 23 eelgrass populations ranging from the Northeast United States to Atlantic, subarctic and Pacific Canada. We identified over 500,000 SNPs, which when mapped to a chromosome‐level genome assembly revealed six broad clades of eelgrass across the study area, with pairwise *F*_ST_ ranging from 0 among neighbouring populations to 0.54 between Pacific and Atlantic coasts. Genetic diversity was highest in the Pacific and lowest in the subarctic, consistent with colonization of the Arctic and Atlantic oceans from the Pacific less than 300 kya. Using redundancy analyses and two climate change projection scenarios, we found that subarctic populations are predicted to be potentially more vulnerable to climate change through genomic offset predictions. Conservation planning in Canada should thus ensure that representative populations from each identified clade are included within a national network so that latent genetic diversity is protected, and gene flow is maintained. Northern populations, in particular, may require additional mitigation measures given their potential susceptibility to a rapidly changing climate.

## 1 INTRODUCTION

Seagrasses and other biogenic habitats are cornerstones of marine conservation, providing numerous ecosystem functions and services. These include nutrient cycling, sediment stabilization and significant carbon storage, in addition to the provision of complex three‐dimensional habitat that supports fisheries and diverse food webs (Nordlund et al., 2016). Seagrasses, including *Zostera marina*, are typically found in coastal nearshore environments in soft‐sediments, located at the interface between land and open ocean. Seagrass meadows are threatened by numerous stressors, including pollution, coastal development, disease and climate change (Leblanc et al., 2023; Martínez‐Abraín et al., 2022; Murphy et al., 2021), contributing to the accelerated loss of global seagrass coverage over the last few decades (Dunic et al., 2021; Waycott et al., 2009). In Canada's marine bioregions, eelgrass (*Zostera marina*) is the most widely distributed seagrass species, occurring in all three bordering oceans (Atlantic, Arctic and Pacific oceans; Murphy et al., 2021).

Across its North American range, eelgrass varies drastically in its form and associated communities, linked with its evolutionary history of colonizing the Atlantic Ocean from the Pacific via the Arctic Oceans approximately 243 kya (Duffy et al., 2022; Yu et al., 2023). Eelgrass can reproduce sexually through dispersal and germination of seeds, and asexually through clonal growth, which impacts its genetic diversity within and between meadows (Kim et al., 2019). Seagrasses can disperse potentially large distances through buoyant reproductive shoots transported by ocean and surface currents and by egestion from vertebrate consumers of seagrasses including fish and waterfowl (Harwell & Orth, 2002; Sumoski & Orth, 2012). Genetic inter‐ and intraspecific variation can affect phenotypic variability and subsequently resilience to environmental change and disturbance (Ehlers et al., 2008; Hughes & Stachowicz, 2004; Phair et al., 2020), and also interact with physical drivers and human stressors to profoundly affect eelgrass density, morphology, resiliency and provision of ecosystem services (Krumhansl et al., 2021; Murphy et al., 2021). Yet climate change is predicted to shift eelgrass distributions northward by ~1.4 to 6.4° by 2100 under different emissions scenarios, with substantial loss of habitat predicted in the southeast and mid‐eastern United States (Wilson & Lotze, 2019). Although projected declines are widespread, some areas may gain suitable habitat from warming temperatures, particularly in northern regions such as James Bay and other parts of Hudson Bay (Wilson & Lotze, 2019). However, in contrast to these predictive models, recent, massive declines in eelgrass extent, density and biomass have been described by Cree land users in James Bay, with limited recovery since the 1990s (Leblanc et al., 2023).

Population genomic diversity and variation associated with the surrounding environment can provide important insights into contemporary distributions and resilience capacity to climate change and other stressors at a population level (e.g. Layton et al., 2021; Plaisted et al., 2020; Stanley et al., 2018). Genomic data can provide insight into population structure and gene flow and permit the detection of genomic regions associated with fine‐scale environmental gradients. Previously, microsatellites have revealed evidence of both fine‐ (i.e. bay‐scale) and broad‐scale (i.e. inter‐continental) population structure in *Z. marina* (e.g. Allcock et al., 2022; Duffy et al., 2022). In northern California, eelgrass shows significant genetic differentiation both within (<5 km apart) and between bays (~23 km), and while clonal richness and heterozygosity did not vary significantly among beds, genetic diversity is often high within beds (Kamel et al., 2012). In fact, eelgrass meadows can be genetically heterogeneous even at the centimetre scale, showing a complex mosaic of genotypes over a small spatial scale (Kollars et al., 2022). Eelgrass beds along coastal South Korea display remarkably high genetic differentiation at eight microsatellites, with *F*_ST_ values (i.e. proportion of total genetic variance in the subpopulation) ranging from 0.061 to 0.573, indicating a strong isolation‐by‐distance (IBD) relationship and limited connectivity (Kim et al., 2017). At the ocean basin and continental scale, eelgrass populations on the Pacific and Atlantic coasts of North America are more genetically similar than either coast is with populations in the Northeast Atlantic (Duffy et al., 2022; Olsen et al., 2004). James Bay in the Canadian subarctic showed affinities with Atlantic populations, and in Europe northern and southern locations form separate, well‐resolved clades (Olsen et al., 2004). Some meadows also show evidence of temporal changes in genetic diversity, likely a result of a changing environment or stressors (Allcock et al., 2022). While these studies revealed differing scales of divergence among eelgrass beds using a few microsatellite loci, applying a high‐throughput sequencing approach to scan the genome for thousands of both candidate adaptive and neutrally evolving markers would provide more accurate metrics of genetic diversity, gene flow and putative environmental adaptation.

Pooled whole‐genome re‐sequencing (poolseq) is a cost‐effective method for obtaining population allele frequencies from pooled individuals (Schlötterer et al., 2014). Poolseq relies on a high‐quality reference genome to align reads and detect genetic variants, and high sequencing depth to distinguish rare alleles from sequencing errors. This method is comparatively less costly than individual‐level genotyping owing to reduced library preparation costs, although data at the individual level is lost. Nevertheless, it is possible to measure robust and unbiased genetic differentiation among populations with poolseq if enough individuals are sampled (Hivert et al., 2018). The first draft of the *Z. marina* genome (assembly size 203 Mb) revealed the molecular mechanisms underlying adaptations for the angiosperm transition from land to the marine environment, including the loss of stomata and adaptations for homeostasis (Olsen et al., 2016). A more recent chromosome‐level assembly using long‐read sequencing improved the assembly length to 260.5 Mb across six chromosomes and several unmapped scaffolds, with 21,483 annotated genes (Ma et al., 2021). Poolseq has been successfully applied in other seagrasses such as *Z. capensis* (Phair et al., 2019, 2021) using the *Z. marina* genome as a reference, with applications in habitat modelling and conservation.

To conserve distinct and isolated populations of *Z. capensis*, Phair et al. (2021) integrated various measures of genomic diversity extrapolated across geographic space with habitat type for conservation planning, finding that genetic variation was not captured by representativity of habitat alone. Phair et al. (2021) concluded that omitting unique populations from the regional Marine Protected Area network could lead to the loss of evolutionarily significant populations and ultimately lead to reduced resilience across its range. To complement work which evaluates representativity and conservation of genetic diversity, analyses that associate environmental conditions with genetic markers can be leveraged to predict the genomic vulnerability or offset (a measure of potential maladaptedness to future climate projections) of a species to predicted environmental change (Nielsen et al., 2021; Rellstab et al., 2021). This information can be critical for conservation planning when prioritizing protection across a species' range. For example, in the seaweed *Phyllospora comosa*, a small panel of single‐nucleotide polymorphisms (SNPs) revealed a higher genomic offset under climate change in the centre of its Australian range despite the relatively high genetic diversity in this area, suggesting these populations might be the most vulnerable to changing environmental conditions (Wood et al., 2021).

Here, we examine genetic diversity and fine‐scale environmental correlations with population structure in eelgrass across eastern North America, the Canadian subarctic (James Bay) and Pacific Canada using poolseq. We focus on identifying detailed population structure and using modelled environmental data to predict population vulnerability to future climate change (i.e. genomic offset; Rellstab et al., 2021) to help inform coastal conservation area planning. We use the chromosome‐level genome assembly from Ma et al. (2021) to detect variants among populations, which is approximately 60 Mb larger than the first *Z. marina* genome assembly (Olsen et al., 2016) and includes information on genomic position of SNPs. Eelgrass in Canada is currently managed as a single phyloregion (Daru et al., 2017), despite evidence for genetic differences among the Atlantic, Pacific and Arctic Oceans (Murphy et al., 2021; Olsen et al., 2004). Poolseq has the potential to reveal fine‐scale population structure, connectivity and both neutral and putatively adaptive evolutionary processes to identify unique eelgrass populations and consider gene flow within conservation planning.

## 2 MATERIALS AND METHODS

### 2.1 Sample collection

**FIGURE 1:**
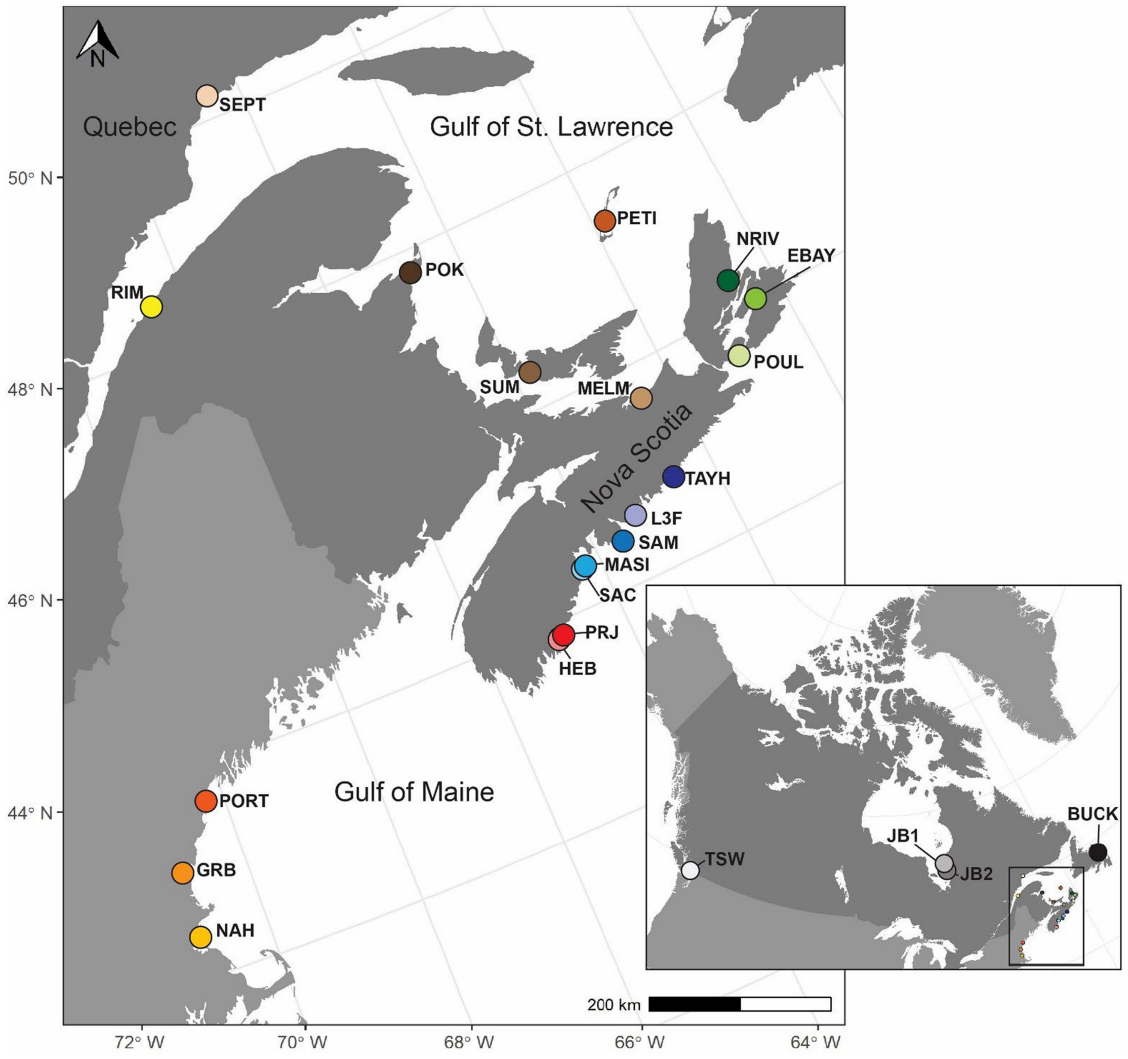
Map of the 23 sampling sites for this study, including Pacific, James Bay and Atlantic sites in Canada and the northeast United States. Inset shows sampling sites outside of the focal Atlantic region. See Table S1 for list of location names.

Eelgrass shoots were collected at 23 sites in 2021 spanning Massachusetts, USA, to Atlantic Canada, James Bay in the Canadian subarctic and one Pacific coast site in British Columbia (Figure 1). The majority of sampling locations were between 1 and 3 m deep, with the exception of Tsawwassen (TSW), Rimouski (RIM) and Sept‐Îles (SEPT), which are intertidal at low tide, and Lower Three Fathom, Nova Scotia, which is a poorly flushed lagoon and typically <1 m (Table S1). At each site, between 30 and 50 vegetative shoots were collected by snorkelling or scuba diving. Individuals were collected at least 2 m apart across approximately the same depth to minimize sampling clonal individuals. In the laboratory, the samples were patted dry and leaves were cut into multiple 5‐cm‐long sections, which were dried with silica beads. Samples were dried for a minimum of 2 weeks prior to DNA extraction.

### 2.2 DNA extraction and pooled sequencing

DNA extraction, library preparation and sequencing were conducted at Génome Québec (Montréal, Canada). Ten milligram of dried eelgrass tissue was added to a microtube plate with a 3 mm tungsten bead, then frozen overnight at −80°C. The Qiagen TissueLyser was used to disrupt the tissue for 1 min at 30 Hz, followed by another 1 min at 30 Hz with the plate reversed. A modified version of the Qiagen DNeasy 96 Plant kit was used for extraction; the chemical lysis was as the protocol describes but the supernatant was filtered with a Qiagen Turbo Filter plate on top of the S‐block by centrifugation at 5 min 6000 *g*. The supernatant was then incubated for 2 h at room temperature. The rest of the extraction was automated using the Qiagen Cube HT with the DNeasy 96 Plant program. The final gDNA elution per individual sample was 100 μL.

gDNA was quantified using the Quant‐iT™ PicoGreen® dsDNA Assay Kit (Life Technologies). Individual eelgrass DNA samples were pooled equimolarly by their collection site prior to normalization for population‐level genome sequencing. Libraries were generated using the NEBNext Ultra II DNA Library Prep Kit for Illumina (New England BioLabs) as per the manufacturer's recommendations. Adapters and PCR primers were purchased from IDT. Size selection of libraries containing the desired insert size of 430–530 bp was performed using SparQ beads (Qiagen). Libraries were quantified using the Kapa Illumina GA with Revised Primers‐SYBR Fast Universal kit (Kapa Biosystems). Average size fragment was determined using a LabChip GX (PerkinElmer) instrument.

The libraries were normalized and pooled followed by denaturing in 0.05 N NaOH and neutralized with HT1 buffer. The pool was loaded at 225 pM on an Illumina NovaSeq6000 S4 lane using the Xp workflow as per the manufacturer's recommendations. The run was performed for 2 × 100 cycles (paired‐end mode). A phiX library was used as a control and mixed with each library at 1% level. Base calling was performed with RTA v3.4.4. The Illumina bcl2fastq2 v2.20 software was then used to demultiplex samples and generate fastq reads.

In addition, 132 individuals from 6 of the 23 sampling sites were genotyped at six multiallelic microsatellites (GA2, GA3, GA4, GA5, GA6, and CT3; Reusch et al., 1999) which revealed relatively few clonal individuals present, and as such pooling individuals for whole‐genome sequencing should provide relatively accurate allele frequencies and heterozygosity estimates. However, it remains unknown how the presence of some clones, particularly in the James Bay sites, when pooled into populations may have impacted nucleotide diversity and Tajima's *D* estimates in our study.

### 2.3 Genome alignment and detecting polymorphisms

The quality of the raw pooled sequences was assessed using FastQC (Andrews, 2010). Reads were quality filtered and trimmed for adapters using fastp (Chen et al., 2018) with a minimum Phred score of 20 and otherwise default parameters. The trimmed reads from each pool were aligned to a publicly available *Z. marina* reference genome assembly (Zostera genome v.3.1; Ma et al., 2021) using the Burrows–Wheeler Aligner bwa‐mem2 2.0 (Vasimuddin et al., 2019) with a minimum mapping quality set to 20 and then converted into sorted binary sequence alignment (bam) files using SAMtools 1.13 (Li et al., 2009). Following the Genomic Analysis Toolkit (GATK 3.7) best practices workflow (DePristo et al., 2011), duplicate reads from library preparation and optical duplicates from sequencing errors were marked and removed using the PicardTools v2.26.1 MarkDuplicates function. Deduplicated reads were then coordinate sorted, indexed and realigned around potential insertions and deletions (indels) using the RealignerTargetCreator and IndelRealigner functions in GATK 3.7. The aligned read depths per pool were assessed in 10 kb windows using mosdepth 0.3.3 (Pedersen & Quinlan, 2018).

The resulting realigned bam files were converted to a mpileup file per pool using the SAMtools mpileup function and then converted to a synchronized file using mpileup2sync.jar in PoPoolation2 (Kofler et al., 2011). All bam files were also combined into a single sync file using the same methods to allow for pairwise comparisons among pools. Allele frequency differences for all populations were computed using the snp‐frequency‐diff.pl function with the combined sync file as input.

### 2.4 Genomic diversity and population structure

Nucleotide diversity (π) and Tajima's *D*, a metric which describes whether a DNA sequence (in this case, the aligned SNPs) is evolving neutrally or through a non‐random process such as selection, were calculated per pool using individual mpileup files and the Variance‐sliding.pl function in PoPoolation v1.2.2, with the following parameters: –measure pi –min‐count 2 –min‐coverage 20 –max coverage 500 –window‐size 10000 –step‐size 10000. While calculating π based only on variant sites (and not the entire genome, including invariant sites) can result in strong downward bias (Korunes & Samuk, 2021), we use this metric simply as a comparative statistic among population by computing π for every pool separately. Next, we calculated *F*_ST_ (i.e. proportion of total genetic variance in the subpopulation) for every individual SNP and windows of SNPs using the fst‐sliding.pl script in PoPoolation2 with the following parameters: ‐‐min‐count 4 ‐‐min‐coverage 20 ‐‐max‐coverage 500 –pool‐size 10 ‐‐window‐size 1000 –step‐size 1000 ‐‐suppress‐noninformative to calculate *F*_ST_ in 1000 window blocks, and with –window‐size 1 and –step‐size 1 to calculate *F*_ST_ for every SNP. Differences in allele frequencies were also calculated in PoPoolation2 using the snp‐frequency‐diff.pl function with the same coverage parameters used previously.

The sync file was converted to pooldata format using the popsync2pooldata function in the *poolfstat* R package, using a minimum coverage of 20, maximum coverage of 500 and a minor allele frequency of 0.05 (Gautier et al., 2022). Pairwise‐*F*_ST_ was calculated among pools using the compute.pairwiseFST function using the ANOVA method with a minimum coverage of 20 and maximum coverage of 500 in *poolfstat*. The isolation‐by‐distance (IBD) relationship was investigated using a Mantel test on a matrix of Slatkin's linearized pairwise *F*_ST_ (Slatkin, 1995) with both Euclidean geographic distance and least‐cost paths through the ocean among sampling sites calculated in the *gdistance* R package (van Etten, 2017). The Pacific (TSW) population was removed from the IBD analysis due to its large distance from all other sites.

Population structure was also investigated using population allele frequencies in the R package *pcadapt* v.4.3.3 (Privé et al., 2020). Hierarchical principal component analyses were conducted on the full set of sampling sites, an Atlantic‐only subset (i.e. Pacific and James Bay removed) and another subset of Nova Scotia populations where the densest field sampling occurred. The number of principal component axes retained was determined using the scree plot for each subset of populations.

The pooldata file was converted to BayPass input using the *poolfstat* pooldata2genobaypass function. BayPass v.2.3 (Gautier, 2015) was used to create a covariance matrix of allele frequencies (Ω) among all populations for pooled data under the core model, using the ‐poolsizefile flag and ‐d0yij (an initial value delta for the initial allele count distribution for the populations) set to 10. A total of 100 pilot runs of 500 iterations were used, followed by a burn‐in period of 5000 iterations. The final Markov chain Monte Carlo sampling for 20,000 iterations was thinned every 20 iterations for 1000 overall parameter values sampled. The resulting covariance matrix was converted into a correlation matrix using the cov2cor R function, and then into a dissimilarity matrix which was visualized as a hierarchical clustering tree using *ape* 5.5 (Paradis & Schliep, 2019).

Finally, Treemix v.1.13 (Pickrell & Pritchard, 2012) was used to assess possible migration scenarios and evolutionary history among all 23 pools. Separate maximum‐likelihood trees were built using 0 through 23 migration events and blocks of 1000 SNPs to account for possible physical linkage among SNPs. The best migration scenario was assessed by its log‐likelihood value and distributions of the model residuals.

### 2.5 Environmental associations and genomic offset

#### 2.5.1 Redundancy analyses

Environmental associations with allele frequencies as the response variable were analysed using redundancy analyses (RDA) that included all sites (excluding TSW due to its high genetic divergence and EBAY where environmental data were unavailable) and for a subset of seven sites in Nova Scotia where finer‐scale environmental data, including substrate composition and growing degree days, were available. For the RDA of the seven Nova Scotia sites, environmental variables important for eelgrass occurrence (see Krumhansl et al., 2021) were measured or calculated for each site which included a Relative Wave Exposure Index (REI), per cent sand in sediments and various metrics of temperature derived from annual in situ time‐series data (RDA_NS‐ENV_). The temperature data used were restricted between June 15 and September 15 (i.e. the main growing season) for each year 2017–2021, with the seasonal mean of each metric used in the analysis. An RDA using 413,551 allele frequencies as the response and the non‐correlated (*r* < 0.85) environmental predictors were conducted using the rda function in the *vegan* R package (Oksanen et al., 2022). Final temperature metrics included maximum temperature, proportion of time spent within the optimal thermal range (i.e. 5 and 23°C) and maximum growing degree day (GDD, i.e. heat accumulation) (Wong & Dowd, 2023). A partial RDA controlling for geography (latitude and longitude) was also conducted (RDA_NS‐COND_), as was an RDA using only geography and no climate variables (RDA_NS‐GEO_) to examine the proportion of total genetic variance in each set of predictors explained. The significance of each predictor was assessed using an ANOVA‐like permutation test with 1000 permutations, with an adjusted *R*‐squared for each model also calculated using 1000 permutations.

For the RDA on the full dataset, 21 of the 23 populations were included (TSW in the Pacific and EBAY in Cape Breton Island were excluded because they were outside the domain of the oceanographic model used). Only loci mapped to the six chromosomes in the assembly were used in the RDAs, while those on unmapped scaffolds were removed, resulting in the inclusion of 393,422 SNPs common to these 21 populations as the response variable. Environmental variables for each sample site were derived from monthly model outputs of surface and bottom salinity and temperature predicted for the North Atlantic between 2008 and 2017 using the Bedford Institute of Oceanography North Atlantic Model (BNAM; Wang et al., 2018), which is based on version 2.3 of NEMO (Nucleus for European Modelling of the Ocean). Data layers were the average of the multi‐year predictions and were converted to an ASCII grid with a NAD83 projection (ellipse GRS80) and a nominal resolution of 1/12° (~5 km^2^) which is uniform between the offshore and inshore. The mean, minimum and maximum seasonal and annual values of temperature and salinity were extracted from these layers for each sample site, and variance inflation factors were used to remove highly correlated variables. The final environmental predictors available from BNAM and used in the RDA_FULL‐ENV_ were mean winter bottom temperature, summer maximum surface temperature, mean spring temperature and annual bottom salinity. Annual salinities ranged from approximately 14 ppt in James Bay to 31–32 ppt in most other sites, while seasonal temperatures ranged from a low of −1.5°C in winter to 21°C in summer, which are all within the known tolerances of eelgrass (e.g. Plaisted et al., 2022). Similar to the RDA for the subset of Nova Scotia populations, a partial RDA using only latitude and longitude as predictors (RDA_FULL‐GEO_) and a partial RDA controlling for geography (RDA_FULL‐COND_) were also run. All environmental variables were centred prior to use in the models.

#### 2.5.2 Detecting outlier loci and climate change projections

To further examine the potential relationship between population structure and environmental and spatial heterogeneity, we identified putative outlier loci using the environment‐only RDA analysis described above (Capblancq & Forester, 2021; Forester et al., 2018; https://github.com/Capblancq/RDA‐landscape‐genomics). The relevant loci were selected based on the extremeness of the Mahalanobis distance estimated between each locus and the centre of the RDA space using the first two RDA axes. These distances were transformed into *p*‐values using a chi‐squared distribution, and loci with false discovery rate corrected *p*‐values <0.05 were identified as candidate outliers (Capblancq et al., 2018). To account for confounding effects of neutral population structure, we also conditioned the environmental RDA on the population ordination scores of the first two axes of the allele frequencies PCA and selected outliers with the same *p*‐value transformation.

A subsequent RDA_OUTLIER_ was then run using the identified candidate outlier allele frequencies as the response variable with the same four environmental predictors for RDA_FULL‐ENV_ previously described to create a spatial projection index of the relative location of each population along RAD axes 1 and 2 (i.e. a representation of population structure associated with low vs. high salinity and low vs. high seasonal temperatures). This index was estimated independently for RDA axes 1 and 2 and was calculated as the sum of each climatic variable score (loading) on the axis multiplied by its standardized value at each focal pixel (Capblancq & Forester, 2021). This index thus shows how allele frequencies correlate with the environmental predictors across the sampling range and illustrates putatively adaptive genomic similarity as a raster. We constrained the raster output to within 20 km of the coastline using a custom shapefile to limit the output of the model to a realistic area where eelgrass can occur.

Genomic offset, or the predicted genomic change necessary for populations to maintain their fitness under a changing environment (Capblancq & Forester, 2021), was then assessed for two global greenhouse gas emissions scenarios (representative concentration pathways (RCP) 4.5 and 8.5) in 2075. RCP4.5 represents a scenario where gas emissions peak in the 2040s and subsequently decline, while RCP8.5 is a scenario where emissions continue to rise unabated. Future projections of temperature and salinity under these RCP scenarios were extracted from BNAM using the same extent and projection as the present climate layers (Wang et al., 2018). A spatial environmental index for each RCP scenario was determined for each informative RDA_OUTLIER_ axis (RDA1 and RDA2), and the genomic offset score was calculated as the difference per pixel between current and future climate adaptive indices. Thus, high scores (i.e. large differences between current and future indices) indicate that a high degree of genetic change is required to persist under that climate scenario. The genomic offset scores for RCP4.5 and 8.5 were then mapped using the same 20 km coastline buffer across the study area.

## 3 RESULTS

### 3.1 Microsatellite genotyping

Six microsatellites were genotyped in 132 individuals in six of 23 populations used for poolseq. The clonal diversity or ratio of unique genets relative to ramets collected, observed and expected heterozygosity and average number of alleles per population are presented in Table S2. Clonal diversity was near 1 in most populations, other than James Bay 1 and 2 (0.73 and 0.6, respectively). These two populations also showed among the lowest observed heterozygosity values and number of alleles, consistent with the presence of some clonal individuals or a population bottleneck. Individuals genotyped at seven other recently collected locations across the Atlantic not used in this study showed high clonal diversity values and thus low presence of clones, indicating our sampling strategy was relatively successful in avoiding clonal individuals (F. Dufresne, unpublished data). Based on our results, we expect that more unique individuals than clones were included in our poolseq analyses, but we cannot rule out the presence of some clones in the James Bay populations, potentially influencing nucleotide diversity and *F*_ST_ values.

### 3.2 SNP filtering and diversity

Overall, 1069 individuals were individually extracted and divided into their respective 23 pools, ranging from *n* = 30 to *n* = 54 individuals. A total of 2,788,570,683 reads were generated across all 23 pools (Table S1). For all pools, >97% of reads mapped to the reference genome with coverage for >75% of the bases ranging from 50 to 100× (Figure S1). After removing duplicate reads ($\bar{x}$ = 21.54% reads per pool) and identifying biallelic markers, *poolfstat* was used to discover 516,094 SNPs with a minor allele frequency > 0.05 from across the genome (Figure S2). These biallelic SNPs and further subsets were used for downstream analyses. Mean genome‐wide nucleotide diversity (π) ranged from 0.67 × 10^−3^ in James Bay to 4.88 × 10^−3^ in the Pacific, with the Atlantic showing intermediate π values (Table S1). Average genome‐wide Tajima's *D* values were negative for all populations and ranged from −0.93 in TSW to −2.08 in HEB, indicating that populations may be approximately at equilibrium or possibly expanding following a geologically recent bottleneck (Yu et al., 2023).

### 3.3 Population structuring

Pairwise *F*_ST_ among populations ranged from 0 between PRJ and HEB, Nova Scotia, to 0.54 when comparing the Pacific to Atlantic populations (Table 1). An isolation‐by‐distance analysis revealed significant positive correlations between linearized *F*_ST_ with both Euclidean straight‐line distance and least‐cost paths following the coastline (*r* = 0.80 and *r* = 0.81, respectively, *p* < 0.001 for both) (Figure S3). Populations that deviated from a simple IBD relationship include RIM, JB1 and JB2 showing a higher‐than‐expected pairwise *F*_ST_, specifically relative to the southernmost sites in Nova Scotia and the northeast United States (0.25–0.33), but a lower‐than‐expected *F*_ST_ between James Bay and Gulf of St. Lawrence populations (0.11–0.15) relative to the coastal distance among them (Figure S3).

**TABLE 1:** Pairwise *F*_ST_ values among all populations based on 516,094 genome‐wide SNPs.

	MASI	SAC	L3F	SUM	POK	PRJ	SAM	NAH	RIM	SEPT	GRB	HEB	PORT	PETI	NRIV	EBAY	POUL	JB1	JB2	BUCK	MELM	TAYH	TSW
MASI	–																						
SAC	0.0024	–																					
L3F	0.0206	0.0189	–																				
SUM	0.1036	0.1021	0.0818	–																			
POK	0.1002	0.0986	0.0805	0.0201	–																		
PRJ	0.0387	0.0376	0.0306	0.1114	0.1081	–																	
SAM	0.0262	0.0219	0.0032	0.0907	0.0889	0.0339	–																
NAH	0.0740	0.0705	0.0639	0.1404	0.1365	0.0862	0.0609	–															
RIM	0.1765	0.1724	0.1529	0.1204	0.1066	0.1787	0.1559	0.2069	–														
SEPT	0.0971	0.0904	0.0733	0.0940	0.0847	0.1001	0.0731	0.1341	0.1294	–													
GRB	0.0819	0.0825	0.0737	0.1479	0.1445	0.0853	0.0766	0.0668	0.2296	0.1428	–												
HEB	0.0343	0.0329	0.0258	0.1081	0.1055	0.0000	0.0307	0.0826	0.1742	0.0985	0.0814	–											
PORT	0.0775	0.0762	0.0694	0.1410	0.1379	0.0828	0.0715	0.0621	0.2161	0.1356	0.0596	0.0787	–										
PETI	0.0672	0.0638	0.0486	0.0331	0.0238	0.0756	0.0547	0.1085	0.1159	0.0672	0.1196	0.0736	0.1100	–									
NRIV	0.0854	0.0852	0.0655	0.0815	0.0759	0.0939	0.0747	0.1355	0.1567	0.0964	0.1404	0.0884	0.1329	0.0535	–								
EBAY	0.0433	0.0425	0.0308	0.0832	0.0800	0.0566	0.0341	0.0900	0.1633	0.0802	0.0996	0.0531	0.0933	0.0497	0.0582	–							
POUL	0.0856	0.0824	0.0650	0.0662	0.0584	0.0936	0.0709	0.1238	0.1397	0.0903	0.1387	0.0907	0.1296	0.0477	0.0537	0.0584	–						
JB1	0.1947	0.1922	0.1685	0.1198	0.1058	0.1992	0.1775	0.2246	0.1771	0.1598	0.2380	0.1947	0.2299	0.1290	0.1798	0.1815	0.1585	–					
JB2	0.1932	0.1910	0.1666	0.1231	0.1100	0.2022	0.1771	0.2172	0.1777	0.1595	0.2423	0.1992	0.2301	0.1269	0.1799	0.1779	0.1514	0.0765	–				
BUCK	0.1042	0.0972	0.0836	0.1129	0.1069	0.1048	0.0826	0.1458	0.1641	0.0672	0.1521	0.1029	0.1465	0.0837	0.1076	0.0890	0.1065	0.1938	0.1976	–			
MELM	0.0721	0.0718	0.0531	0.0331	0.0364	0.0793	0.0604	0.1137	0.1307	0.0773	0.1198	0.0778	0.1109	0.0243	0.0626	0.0511	0.0548	0.1387	0.1405	0.0936	–		
TAYH	0.0264	0.0203	0.0061	0.0871	0.0833	0.0357	0.0052	0.0668	0.1574	0.0689	0.0799	0.0322	0.0713	0.0489	0.0713	0.0311	0.0684	0.1766	0.1760	0.0806	0.0550	–	
TSW	0.4453	0.4430	0.4282	0.4563	0.4593	0.4209	0.4282	0.4572	0.5209	0.4534	0.4754	0.4271	0.4666	0.4471	0.4813	0.4569	0.4758	0.5405	0.5329	0.4628	0.4568	0.4334	–

*Note*: The highest pairwise *F*_ST_ occurs between Atlantic and Pacific sample sites (>0.5), with sites in the subarctic (James Bay) showing intermediate values (~0.1–0.2).

**FIGURE 2:**
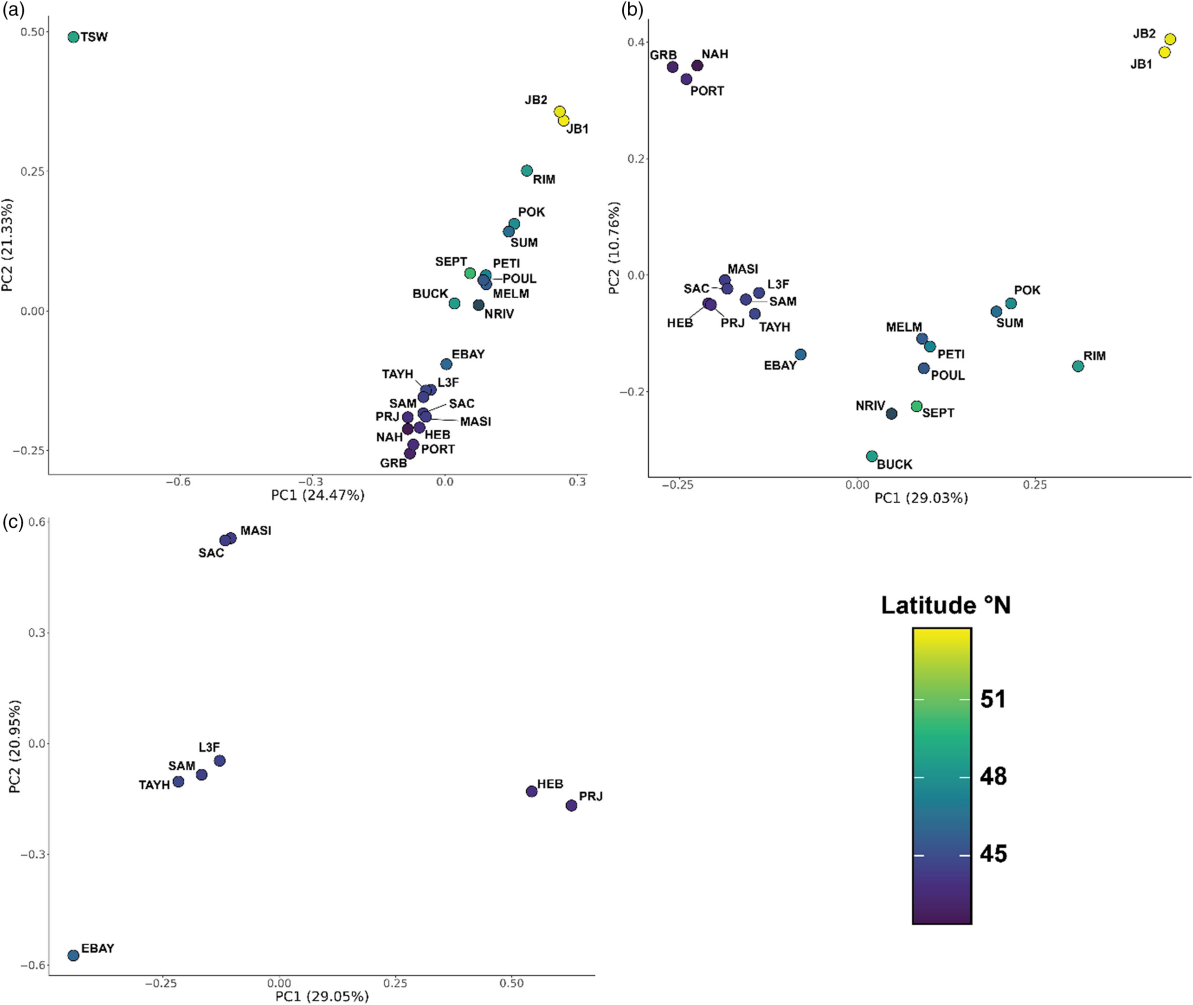
Plots depicting principal components analyses of SNP data based on (a) all sampled sample locations, (b) Atlantic and Arctic samples and (c) coastal Nova Scotia locations.

Principal component analyses allowed for a hierarchical analysis of population structure, which showed the same broad genetic divergence between Pacific and Atlantic/Arctic populations along PC1 (24.47% of the variance), and a general latitudinal gradient along PC2 (21.33%) ranging from the USA to James Bay (Figure 2a). Within just Atlantic and subarctic populations, James Bay populations were the most divergent on PC1 (29.03%) and PC2 (10.76%), while USA, Nova Scotia, Bras D'Or Lake, the Gulf of St. Lawrence and Newfoundland populations formed well‐defined clusters along these axes (Figure 2b). Finally, when only examining coastal Nova Scotia and Bras D'Or Lake, four clusters were defined on PC1 (29.05%) and PC2 (20.95%), namely Bras D'Or Lake (EBAY), Nova Scotia South Shore (HEB and PRJ), Nova Scotia islands (MASI and SAC) and Halifax/Eastern Shore (TAYH, SAM and L3F) (Figure 2c).

**FIGURE 3:**
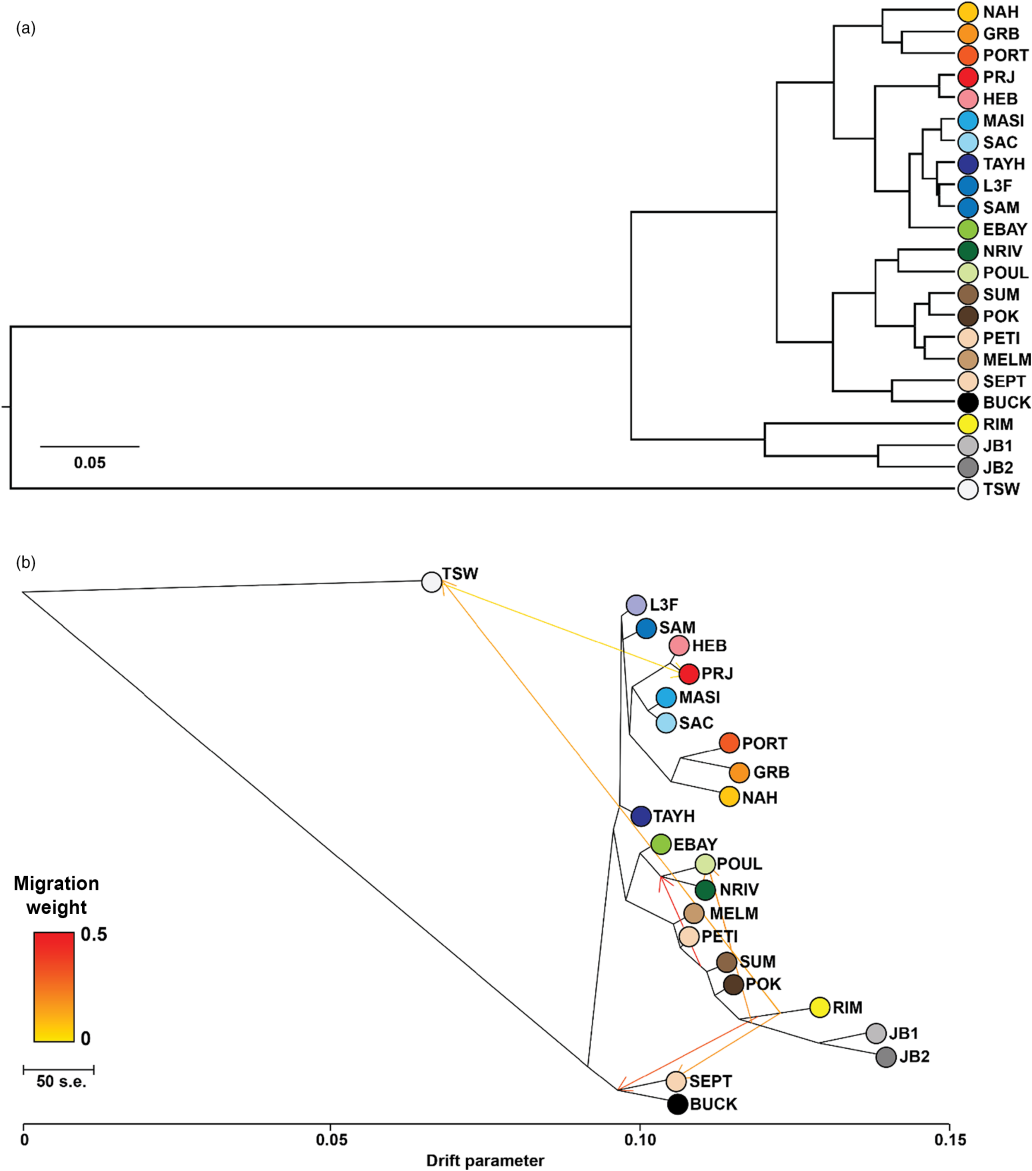
(a) Hierarchical clustering tree based on BayPass covariance matrix. Major divisions include the Pacific site (TSW) as an outgroup to all other populations, James Bay (JB1 & 2) clusters with Rimouski as an outgroup to Atlantic populations, US sites form their own cluster and Atlantic Nova Scotia forms a different cluster than the Gulf and Cape Breton. (b) Maximum‐likelihood tree from Treemix including *m* = 6 migration events.

A hierarchical clustering phylogram based on the population covariance matrix generated by BayPass (Figure S4) shows TSW (Pacific) as the outgroup to all the Atlantic and James Bay samples (Figure 3a). Interestingly, the Rimouski population (RIM) clustered with the two James Bay populations (JB1, JB2) separated by ~4000 km ocean distance rather than Sept‐Iles, which lays ~270 km on the adjacent side of the St. Lawrence Seaway. The SEPT population in turn clustered with Buckley Cove, NL (BUCK). The three populations from the USA (PORT, GRB and NAH) formed a distinct cluster in the Atlantic, while most of Cape Breton (NRIV and POUL) and all the Gulf of St. Lawrence (PETI, SUM, MELM, POK) formed a cluster which was sister to the Atlantic Nova Scotia populations (SAM to PRJ). EBAY, in the semi‐enclosed Bras D'Or Lake in Cape Breton, grouped with the Atlantic Nova Scotia populations, rather than more proximate Cape Breton populations (NRIV and POUL).

Treemix showed the same general structure patterns as BayPass, with TSW showing the lowest relative genetic drift value, and the two James Bay populations showing the highest drift parameter (Figure 3b). However, in Treemix, the EBAY population clusters with other geographically proximate Cape Breton populations (POUL and NRIV) as opposed to the Atlantic Nova Scotia cluster from the BayPass clustering tree. Twenty‐three migration events were supported by the highest log‐likelihood (1903.57 relative to 245.08 for 0 migrations) but primarily showed gene flow among populations within monophyletic clusters. At six migration events (Figure S5), the RIM plus James Bay cluster showed potential migrations to Cape Breton and the SEPT population, as well as migrations to TSW, while TSW showed potential gene flow to the southern Nova Scotia populations. The potential migration events between Atlantic and Pacific populations likely represent the ancient colonization of the Atlantic from the Pacific.

### 3.4 Environmental associations with population structure

The subset of seven coastal Nova Scotia populations showed a global significance with the selected environmental parameters (*p* = 0.007, $R_{\mathrm{adj}}^{2}$ = 0.30; Table 2). A permutation test revealed allele frequencies are significantly associated with gradients of maximum temperature and sediment composition (% sand; *p* < 0.05). The RDA_NS‐ENV_ using the allele frequencies accounted for 88% of the total constrained variance and allowed for clear separation of the study sites. RDA1 accounted for 23.83% of the total variance and was defined primarily by maximum temperature and GDD which had the highest positive loadings, and prop 5.23 which had the lowest negative loadings (Figure S5). This strongly differentiated population structure at PRJ and HEB from the other sites, with PRJ and HEB having the highest maximum temperatures, highest heat accumulation and least amount of time spent in the optimal temperature range. RDA2 explained 21.77% of the variance in population structure across sites and was primarily defined by per cent sand in sediments and wave exposure (highest negative loadings; Figure S6). This differentiated the population structure of exposed and sandy sites on the open coast (SAM and TAYH) from more protected island sandy sites (MASI and SAC) and protected muddy sites at the heads of bays or in lagoons (HEB, PRJ and L3F). The partial redundancy analysis controlling for site latitude and longitude (RDA_NS‐COND_) was not significant (*p* = 0.438), while the pure geographic RDA_NS‐GEO_ was significant at *p* = 0.004.

**TABLE 2:** Summary of redundancy analyses for the Atlantic and subarctic sites (*n* = 21) and a subset of Atlantic Nova Scotia sites (*n* = 7) showing the adjusted *R* ^2^, the *p*‐values and proportion of the variance explained by the model.

Model	$R_{\mathrm{adj}}^{2}$	*p* (*>F)*	Constrained proportion
Nova Scotia			
RDA_NS‐ENV_ Environmental (MaxTemp + REI + GDD5 + prop5.23 + per cent sand)	0.30	0.007	0.88
RDA_NS‐COND_ Environmental + Condition (Lat + Long)	0.01	0.438	0.42
RDA_NS‐GEO_ Lat + Long	0.17	0.004	0.44
Atlantic + Subarctic			
RDA_FULL‐ENV_ Environmental (AnnMeanSalinity + WinterMinimumBTEMP + SpringMeanSST + SummerMaxSST)	0.19	<0.001	0.35
RDA_FULL‐COND_ Environmental + Condition (Lat + Long)	0.10	<0.001	0.24
RDA_FULL‐GEO_ Lat + Long	0.16	<0.001	0.25
Outlier RDA			
RDA_OUTLIER_ Environmental (AnnMeanSalinity + WinterMinimumBTEMP + SpringMeanSST + SummerMaxSST)	0.0.78	<0.001	0.82

**FIGURE 4:**
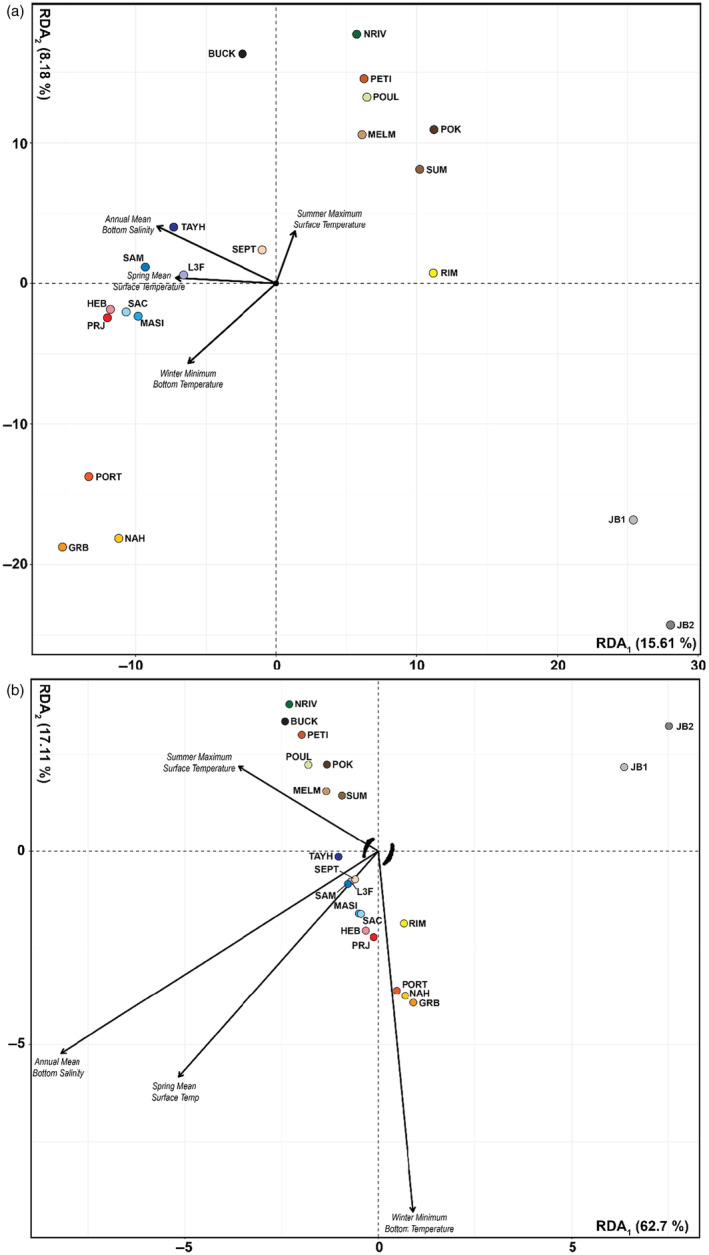
(a) Redundancy analysis of 21 populations. (b) Redundancy analysis for 21 populations using 738 putative outlier SNPs (black points) to calculate adaptive indices along RDA 1 and 2. RDA 1 is primarily driven by annual bottom salinity, while RDA 2 is driven by temperature variation and latitude.

RDA_FULL‐ENV_ of 21 sites using the four seasonal (temperature) and annual (salinity) climate variables explained 35% of the variance in allele frequencies and was globally significant at *p* < 0.001 and $R_{\mathrm{adj}}^{2}$ = 0.19 (Figure 4a, Table 2), as were the RDAs conditioning the environment by geography (RDA_FULL‐COND_) and a purely geographic (RDA_FULL‐GEO_). A total of 738 genome‐wide loci (Figure S7) were selected as putative outliers based on their distribution among the loadings along the first two RDA axes.

**FIGURE 5:**
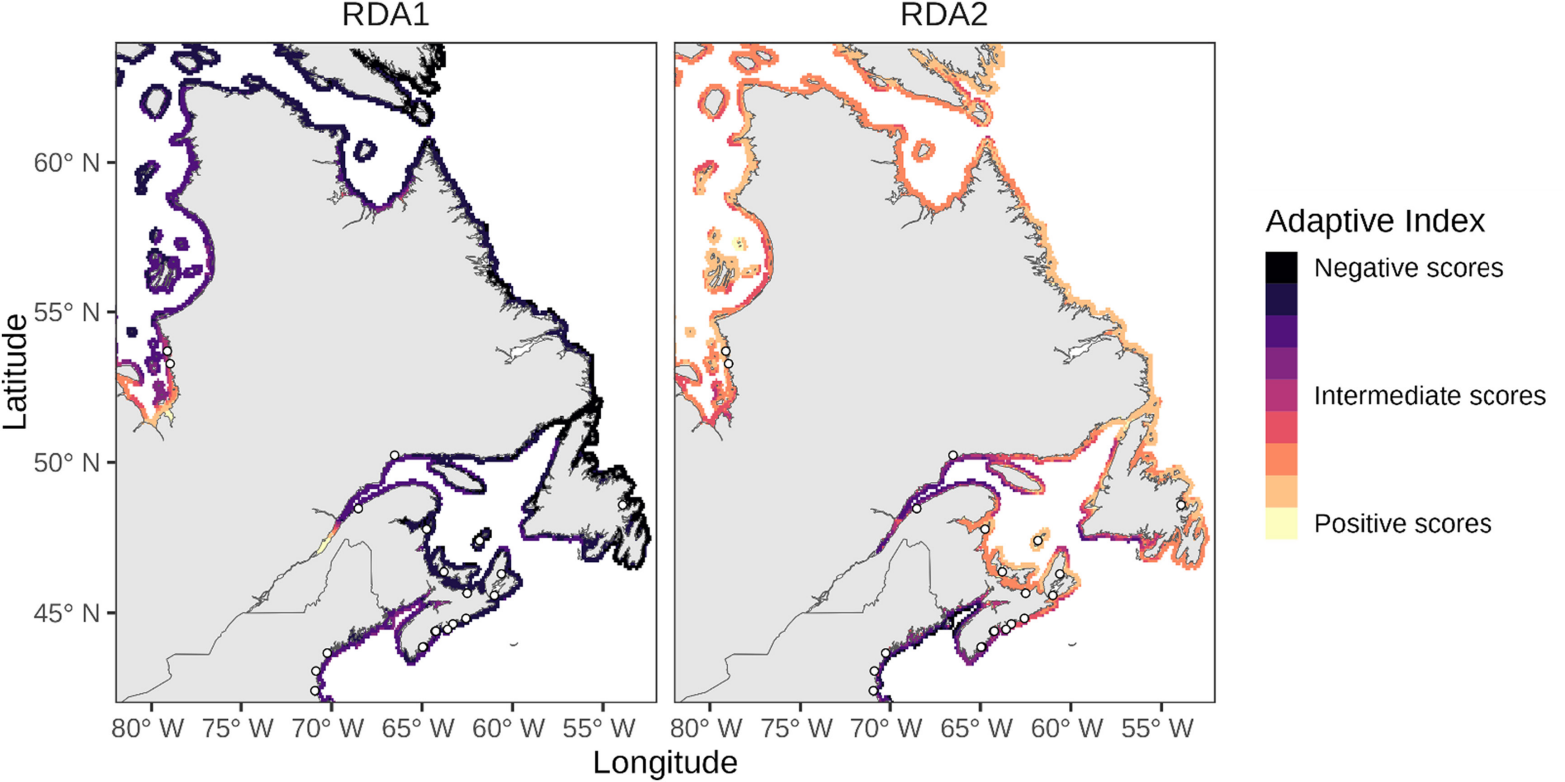
Adaptive index scores sensu Capblancq and Forester (2021) based on 738 putative outlier SNPs detected by redundancy analysis axes (RDA) 1 and 2 (see Figure 4b). RDA 1 is primarily driven by differences in average salinity, and to a lesser extent, spring and summer temperatures, with negative scores corresponding to areas of higher salinity and higher temperature (darker shading). RDA 2 is primarily driven by winter temperature differences associated with latitude, where negative scores are associated with higher minimum winter temperatures and positive scores are associated with colder winter temperatures (lighter shading).

The RDA based on the outlier loci was significant at *p* < 0.001 with an $R_{\mathrm{adj}}^{2}$ = 0.78 and explained 82% of the total constrained variance (Table 2). RDA1 was positively associated with annual mean bottom salinity and, to a lesser extent, negatively associated with summer temperature, explaining 62.70% of the variation (Figure 4b). RDA2 was associated with minimum winter temperature on the negative side of the axis and explained 17.11% of the variance. When projected across the study seascape, the environmental index scores were generally negative on both RDA axes in the southern region of the study area (i.e. the northeast USA and southern Nova Scotia) and generally positive in northern Newfoundland, Labrador and the subarctic (Figure 5). These patterns indicate a genetic gradient associated with differences in bottom salinity and summer temperature (RDA1) and with differences in minimum winter temperature (RDA2) across the seascape (Figure 5).

**FIGURE 6:**
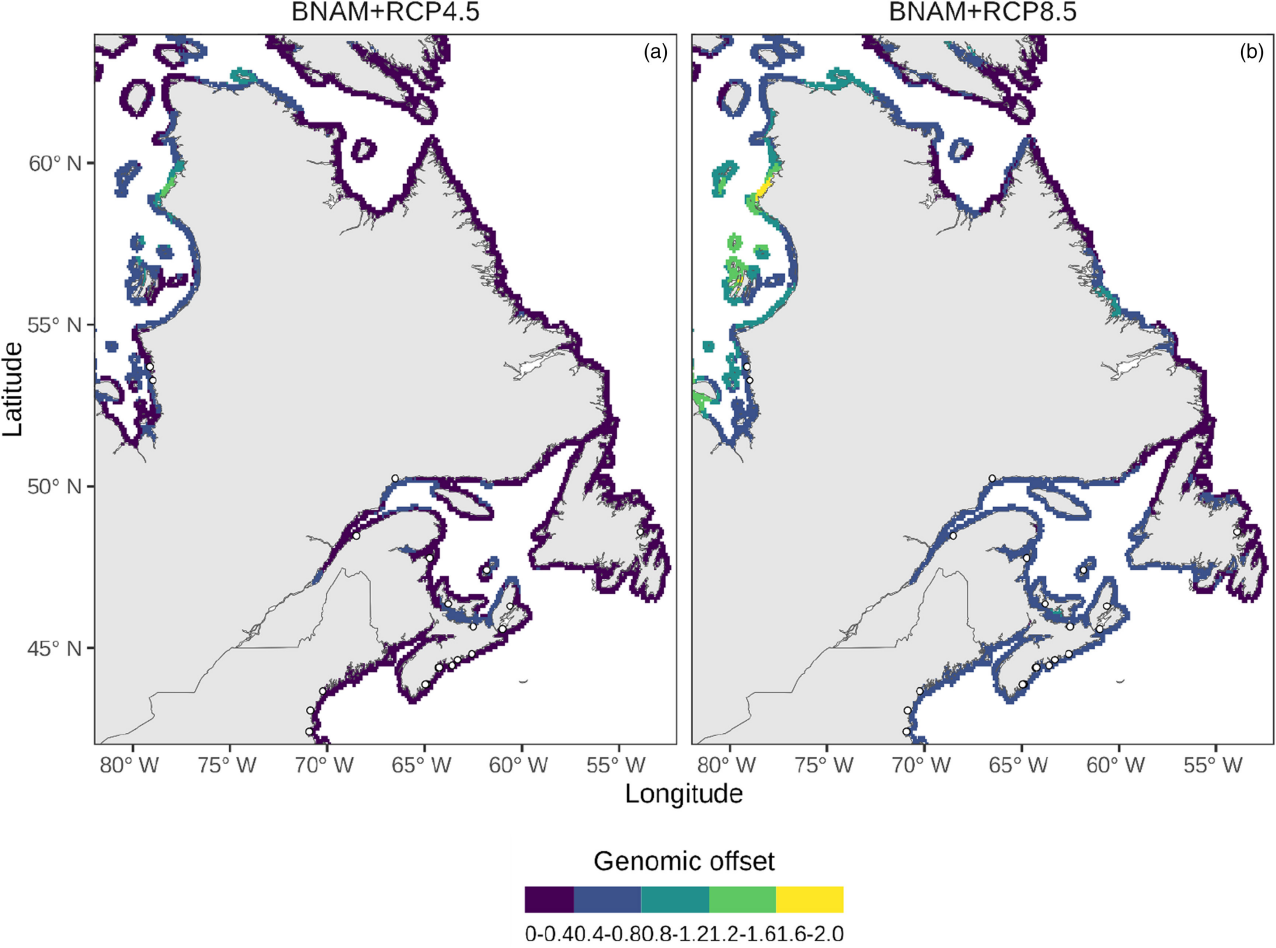
Genomic offset scores for two climate change emissions scenarios (a) representative concentration pathway (RCP) 4.5 and (b) RCP 8.5 combined with the Bedford Institute of Oceanography North Atlantic Model (BNAM). The highest genomic offset scores occur in Hudson Bay and parts of James Bay, especially under RCP 8.5. Intermediate offset scores occur in southern populations and the Gulf of St. Lawrence, while the lowest scores are seen in northern Newfoundland and Labrador.

A principal component analysis of current and projected seasonal temperature and salinity values shows substantial climate change on axis PC2, which is primarily associated with seasonal temperatures (Figure S8). Genomic offset scores were higher under the RCP 8.5 emissions scenario relative to RCP 4.5 as expected, with increased warming associated with higher emissions (Figure 6). The largest offset scores were associated with the subarctic populations in James Bay, especially under RCP8.5. Under RCP 8.5, scores outside of the subarctic were generally intermediate in value, while they tended to be lower under RCP 4.5. Offset scores for populations in the south of the study region (Southern Gulf of St. Lawrence, Nova Scotia and the Gulf of Maine) remained relatively unchanged between emission scenarios (Figure 6).

## 4 DISCUSSION

In Canada, eelgrass is managed as a single widespread phylogroup (Murphy et al., 2021), yet our results reveal significant population structuring among three ocean basins and at regional scales consistent with earlier microsatellite analyses (Olsen et al., 2004). We reveal evidence of genetic structure at several spatial scales, confirming that genetically distinct populations exist, which should be considered when designing coastal conservation areas (Figure 2). Using low‐coverage, whole‐genome sequences for >1000 individuals combined into 23 pools, we detected high pairwise *F*_ST_ (>0.5) between Atlantic and Pacific populations, with James Bay sites in the subarctic showing intermediate values between the Atlantic and Pacific coasts (Table 1). The two subarctic James Bay sites clustered with the Atlantic samples – specifically RIM (Rimouski, QC) – while the Pacific is more distantly related to all the other sites (Figure 3). It is unknown why the James Bay sites were genetically similar to the Rimouski population specifically, although the lower salinity James Bay populations may be derived from a St. Lawrence estuary population via long‐distance seed dispersal in fish or waterfowl, for example (Sumoski & Orth, 2012). Our results support previous studies that used multiallelic microsatellites, where James Bay populations clustered with Atlantic populations, and Pacific populations form the outgroup (although all North American populations are more closely related than they are to Atlantic European populations) (Olsen et al., 2004). Little sequence divergence in the internal transcribed spacer (ITS) region of the genome suggests recent divergence among North American Pacific and Atlantic populations (Olsen et al., 2004), which was later estimated to be ~243 kya based on nuclear and chloroplast gene phylogenies (Yu et al., 2023). Despite clear genomic differences among oceans, there is still a weak signal of Pacific ancestry in southern Nova Scotia populations, as evidenced by the covariance matrix and Treemix migration scenarios. The genetic structuring observed here and in other studies has been found to correspond with differences in phenotype and biomass in the Atlantic and Pacific regions, which has profound impacts on the biodiversity and communities associated with eelgrass beds (Duffy et al., 2022).

Genetic diversity (heterozygosity and allelic richness) has been demonstrated to correlate positively with resilience to stressors such as low light and increased sediment loads in eelgrass (Plaisted et al., 2020, 2022). Differences in genetic diversity among sampled populations as indicated by Tajima's *D* statistic were negative for all populations in our study, potentially indicative of postglacial population expansion or a signal of recent natural selection leading to excessive rare alleles (Dennenmoser et al., 2017). While genotyping a small subset of individuals at six microsatellites revealed clonal diversity values >0.5 in six populations – thus more unique individuals than clones – the lowest clonal diversity, observed heterozygosity and nucleotide diversity values were all observed in James Bay 1 and 2 (Table S2). It is thus possible that these low diversity and heterozygosity values are the result of the presence of some clonal individuals, genetic drift at the northern edge of its range or both. However, our results are consistent with other studies which did not find clones present (Olsen et al., 2004; Reusch et al., 2000; Yu et al., 2023), and thus we believe that the presence of some clonal individuals did not greatly impact our poolseq results. The Pacific site (TSW) had an average T_D_ closest to zero, indicating a population close to equilibrium, while more negative values in the Atlantic and subarctic populations potentially indicate more recent population expansion. TSW also showed approximately four times greater nucleotide diversity (π) compared to all Atlantic sites, while, in contrast, the lowest diversity was found in the subarctic James Bay sites (Table S1). Pacific populations of eelgrass have been shown to have greater genetic diversity than eastern and western Atlantic populations, consistent with evidence that eelgrass first evolved in the Pacific between 10 and 5 million years ago, and subsequently spread to the Atlantic via the Arctic (Duffy et al., 2022; Olsen et al., 2004; Yu et al., 2023). In fact, while we detected substantial population structure in the temperate Atlantic, whole‐genome data from additional locations in the Pacific could be expected to reveal even more structure, as microsatellites have revealed even greater divergence within the Pacific relative to the Atlantic and subarctic (Duffy et al., 2022). The last glacial maximum likely severely reduced genetic diversity in Canadian subarctic/arctic populations, and the reduced diversity observed here is consistent with a recent re‐colonization of the subarctic from a southern refugium and ongoing genetic drift via a bottleneck effect (Kardos et al., 2021). This recent re‐colonization is also supported by the high drift parameter values for James Bay in the Treemix analyses (Figure 3). While omitting missing and invariant sites from genomic diversity calculations can lead to downward bias in these values (Korunes & Samuk, 2021), the sliding window approach used here on genome‐aligned reads should reduce these biases (Kofler et al., 2011). Nevertheless, we use these estimates of genetic diversity for relative comparisons only. Ongoing monitoring of eelgrass using genome‐wide markers and actions to conserve genetic variation among populations is a recommended approach to reduce the negative effects of inbreeding and prevent the loss of adaptive potential (Kardos et al., 2021).

We identified 738 loci as putative outliers (~0.15% of all loci) which are significantly correlated with seasonal ocean temperatures and salinity; however, based on their distribution in the RDA space, these loci are also likely associated with population structure and latitude. While this may lead to the identification of false‐positive outlier loci (Capblancq & Forester, 2021), the patterns in the outlier‐environmental seascapes correspond with known tolerances of salinity and temperature for our study populations, which have measurable fitness impacts when temperatures exceed 25°C or salinities below 15‰ (Nejrup & Pedersen, 2008; Salo et al., 2014). Within Nova Scotia, genetic structure in the southernmost sites was primarily associated with high maximum summer temperatures and heat accumulation, while eastern populations (L3F and TAYH) are associated with relatively higher wave exposure and sandier sediments (Figures 4 and 5, Figure S5; Krumhansl et al., 2020).

Based on loadings of the outlier SNP allele frequencies correlated with the environment on the first RDA axis, southern populations inhabit warmer and higher salinity waters, whereas populations further north are associated with colder temperatures and lower salinity (or other variables that often correlate with salinity such as turbidity and coloured dissolved organic matter). Northern populations also were only collected from lower salinity areas, so there may be a sampling bias that confounds salinity and latitude in these data.

Genomic offset is a prediction of theoretical maladaptiveness, where the scores for regions in between sampling sites are calculated assuming a linear relationship between climate and allele frequencies (Rellstab et al., 2021). Based on future climate predictions under two emissions scenarios, the Canadian subarctic demonstrates the highest genomic offset scores, suggesting they may be the most vulnerable to changing ocean conditions by 2075, particularly under the more extreme RCP8.5 scenario (Figure 6). This may be due to a combination of low genetic diversity and predicted rapid ocean warming in Hudson/James Bay. In the subarctic, an increase in suitable habitat for eelgrass has been predicted to occur with a warming climate (Wilson & Lotze, 2019), yet the extensive meadows in James Bay recently underwent a rapid decline in the late 1990s and have shown limited recovery by 2020 (Leblanc et al., 2023). This decline coincided with anomalously high temperatures and ice break‐up conditions in 1998 during an El Niño event and followed substantial changes in freshwater input associated with hydroelectric development (Leblanc et al., 2023). In another marine macrophyte, the temperate seaweed *Phyllospora comosa*, populations at the leading edge of their range exhibited low diversity, but genomic offset scores were also relatively low (Wood et al., 2021). Wood et al. (2021) propose that rare alleles in leading‐edge populations of *P. comosa* may be important in the survival of the species under climate change through gene flow and assisted evolution. Both the environment–allele frequency index and genomic offset maps should be used with caution for regions where no genetic sampling took place, such as along the Labrador, Ungava Bay and Hudson Bay coasts. At present, the indices mapped for these regions are extrapolations between sampling areas and adding new sampling sites could help validate and refine our genomic offset predictions. Overall, eelgrass is predicted to shift the southern extent of its range around North Carolina at least 1–6° north by 2100 (Wilson & Lotze, 2019), and additional genomic sampling in both the southern and northern limits of *Z. marina* could provide data to monitor latitudinal shifts in genetic diversity and environmental associations with population structure. Additionally, while genomic offset estimates are the result of correlations among allele frequency, the environment and predicted future climate, they can also be validated using experimental methods (Rellstab et al., 2021). For example, in lodgepole pine (*Pinus contorta*), genomic offset scores show a strong, negative relationship with reared seedling fitness (Capblancq & Forester, 2021; Fitzpatrick et al., 2021).

For eelgrass conservation and restoration efforts, it is crucial to understand its genetic diversity and structure, particularly along environmental gradients and across ocean basins (Pazzaglia et al., 2021; Reynolds et al., 2012). For example, if transplanting individuals or spreading seeds across broad geographic ranges to restore degraded habitats, it could be beneficial to consider prioritizing the use of admixed genotypes and/or genotypes with capacity to succeed in predicted future environmental conditions using metrics such as genomic offset, as opposed to necessarily using the most proximate population's genotypes (Pazzaglia et al., 2021; Plaisted et al., 2022). Prioritizing transplantation of populations with higher diversity has also been shown to reduce transplant shock/stress and loss of plants during restoration efforts (Hughes & Stachowicz, 2004). The integration of genetic diversity and genetic connectivity metrics with biogeographical and oceanographic data, such as sedimentology, ocean temperature, salinity, currents and anthropogenic impacts, can help refine species distribution models (Lowen et al., 2019), conservation planning approaches (Phair et al., 2021) and inform propagule exchange and functional connectivity networks (Pazzaglia et al., 2021). Combining our adaptive index maps with a high‐resolution species distribution model (e.g. O'Brien et al., 2022) could provide novel prioritization layers for conservation planning by identifying climate‐resilient populations and connectivity pathways, while also guiding current restoration efforts.

Our results support both broad‐ (1000s km) and fine‐scale (10s km) population structuring in *Z. marina* in North America, with at least six clades across the continent, and an additional four distinct groups detected along a fine‐spatial scale in Nova Scotia where most samples were collected. Conservation of genetic diversity in this ecologically important coastal species necessitates that tools like MPAs and marine spatial planning prioritize representativity and uniqueness of the underlying genetic structure (e.g. Nielsen et al., 2022). By integrating even basic metrics of genomic diversity with habitat suitability models in conservation planning, unique alleles and resilience may be sufficiently protected to allow the long‐term persistence of seagrasses facing numerous stressors (e.g. Phair et al., 2021). Moreover, genomic tools (such as genomic offset analyses) can allow consideration of resilience to environmental change when building networks of conservation areas (e.g. Bryndum‐Buchholz et al., 2022). The pooled sequencing method used here allows the addition of other populations in the future, such as those where sampling effort is low, particularly in the Pacific and Arctic oceans, and along coastal Labrador. Additional environmental predictors could be included in the genome–environment associations, such as sediment composition, wave exposure, dissolved oxygen and stressors such as pollution and coastal development, which may provide a more detailed picture of the genomic seascape for eelgrass.

## FUNDING INFORMATION

This study was funded through a Fisheries and Oceans Canada Competitive Science Research Fund awarded to NJ, BV, RS and MW. Fieldwork in eastern James Bay was supported by the Niskamoon Corporation, a Cree‐run organization and part of the Coastal Habitat Comprehensive Project, which aimed to better understand coastal ecosystems, including eelgrass habitats, in eastern James Bay by partnering with Cree land users (https://www.eeyoucoastalhabitat.ca). We wish to acknowledge Cree land users and community members for authorizing research to be conducted on their traditional hunting territories.

## CONFLICT OF INTEREST STATEMENT

The authors declare no conflict of interest exists.

## Supporting information


Data S1

## Data Availability

Genetic Data: Raw DNA sequence reads and metadata for each pool are deposited in the NCBI Sequence Read Archive at https://www.ncbi.nlm.nih.gov/sra/PRJNA891275. The genome assembly used for read alignment is available at https://data.jgi.doe.gov/refine‐download/phytozome?organism=Zmarina&expanded=Phytozome‐668. Sample metadata and processed data files are deposited in the Dryad Digital Repository. All annotated scripts and metadata for this study are available at https://github.com/NickJeff13/Eelgrass_Poolseq.
