# Supplementary material for: Variation in genomic vulnerability to climate change across temperate populations of eelgrass (Zostera marina)
Source: Evol Appl. 2024 Apr 21;17(4):e13671. doi: 10.1111/eva.13671 (PMC11033490; doi:10.1111/eva.13671)
Supplement: Supplementary file 1 — Data S1 [file EVA-17-e13671-s001.docx]

# Supplementary Information

Supplementary tables and figures, including sample metadata, are included in the supplementary information.

Table S1. The pool numbers and associated sample site codes, the number of reads, percent duplicate reads, and genome-wide mean nucleotide diversity (𝝅) and mean Tajima’s *D* (T*_D_*) for each pool. All samples were collected in 2021.

| **Site** | **Code** | **Bed Type** | **Pool** | **Individuals Pooled** | **Reads** | **% Duplicate** | **Mean 𝝅 (x10^-3)** | **Mean T*_D_*** |
| --- | --- | --- | --- | --- | --- | --- | --- | --- |
| Masons Island, Nova Scotia | MASI | Subtidal | 1 | 47 | 120,934,875 | 20.35 | 0.9362 | -1.65 |
| Sacrifice Island, Nova Scotia | SAC | Subtidal | 2 | 47 | 110,045,009 | 20.56 | 0.9444 | -1.41 |
| Lower Three Fathom, Nova Scotia | L3F | Lagoon shallow subtidal | 3 | 46 | 137,341,765 | 23.36 | 0.9775 | -1.72 |
| Summerside, Prince Edward Island | SUM | Subtidal | 4 | 48 | 208,184,559 | 23.67 | 0.9200 | -2.03 |
| Pokesudie, New Brunswick | POK | Subtidal | 5 | 47 | 155,912,650 | 25.07 | 0.8812 | -1.93 |
| Port Joli, Nova Scotia | PRJ | Subtidal | 6 | 47 | 128,351,651 | 22.07 | 1.095 | -1.96 |
| Sambro, Nova Scotia | SAM | Subtidal | 7 | 47 | 111,055,087 | 21.54 | 0.9927 | -1.50 |
| Nahant, Massachusetts | NAH | Subtidal | 8 | 47 | 110,071,735 | 21.52 | 0.9865 | -1.36 |
| Rimouski, Québec | RIM | Intertidal | 9 | 41 | 106,099,603 | 20.87 | 0.7371 | -1.55 |
| Sept Îles, Québec | SEPT | Intertidal | 10 | 39 | 117,721,459 | 23.20 | 0.9075 | -1.37 |
| Great Bay, New Hampshire | GRB | Subtidal | 11 | 49 | 96,097,373 | 19.05 | 0.8934 | -1.66 |
| Port L’Hebert, Nova Scotia | HEB | Subtidal | 12 | 48 | 238,964,366 | 25.19 | 1.0657 | -2.08 |
| Portland, Maine | PORT | Subtidal | 13 | 47 | 127,494,441 | 22.39 | 0.9454 | -1.36 |
| Petite Baie, Magdalen Islands | PETI | Subtidal | 14 | 47 | 114,810,405 | 20.32 | 0.9011 | -1.68 |
| North River, Cape Breton, Nova Scotia | NRIV | Subtidal | 15 | 47 | 91,904,377 | 19.30 | 0.8247 | -1.42 |
| East Bay, Cape Breton, Nova Scotia | EBAY | Subtidal | 16 | 47 | 97,833,914 | 24.44 | 0.8758 | -1.41 |
| Poulamon, Isle Madame, Nova Scotia | POUL | Subtidal | 17 | 47 | 90,600,215 | 22.24 | 0.8406 | -1.18 |
| James Bay 1 | JB1 | Subtidal | 18 | 30 | 96,877,734 | 20.79 | 0.6697 | -1.36 |
| James Bay 2 | JB2 | Subtidal | 19 | 30 | 97,522,766 | 19.99 | 0.7205 | -1.15 |
| Buckley Cove, Newfoundland | BUCK | Subtidal | 20 | 54 | 132,664,702 | 20.53 | 0.8869 | -1.42 |
| Melmerby, Gulf of St. Lawrence, Nova Scotia | MELM | Subtidal | 21 | 50 | 104,079,936 | 22.29 | 0.8913 | -1.48 |
| Taylor Head Provincial Park, Nova Scotia | TAYH | Subtidal | 22 | 40 | 107,618,813 | 18.44 | 0.9537 | -1.54 |
| Tsawwassen Beach, British Columbia | TSW | Intertidal | 23 | 30 | 86,383,248 | 18.31 | 4.8792 | -0.93 |

Table S2. The results from genotyping individuals at six microsatellites (GA2, GA3, GA4, GA5, GA6, CT3; Reusch et al. 1999) from six populations from the current study, including clonal diversity, observed and expected heterozygosity, and the average number of alleles per population. Clonal diversity for all six populations is >0.5 which indicates more unique individuals than clones. The observed heterozygosity and number of alleles is lowest in the James Bay populations, which may indicate the presence of some clones, population bottlenecks, or both.

| Populations | Sample size (N) | Multilocus  genotypes | Clonal  Diversity (R) | Observed  heterozygosity | Expected  heterozygosity | Number of alleles |
| --- | --- | --- | --- | --- | --- | --- |
| Rimouski (QC) | 28 | 27 | 0.96 | 0.364 | 0.420 | 4.50 |
| Sept-Iles (QC) | 30 | 28 | 0.93 | 0.456 | 0.477 | 4.50 |
| James Bay 1 | 23 | 17 | 0.73 | 0.217 | 0.254 | 2.333 |
| James Bay 2 | 24 | 15 | 0.6 | 0.303 | 0.456 | 2.833 |
| Lower three  Fathom (NS) | 29 | 29 | 1.0 | 0.561 | 0.582 | 4.833 |
| TSW (BC) | 17 | 16 | 0.94 | 0.282 | 0.445 | 5.333 |


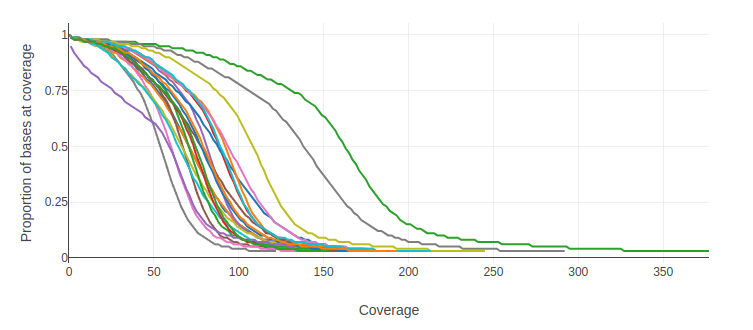


Figure S1. Sequencing coverage for each of the 23 pooled populations, indicated by different line colours. More than 75% of each pool’s sequenced bases have a depth >50X coverage.


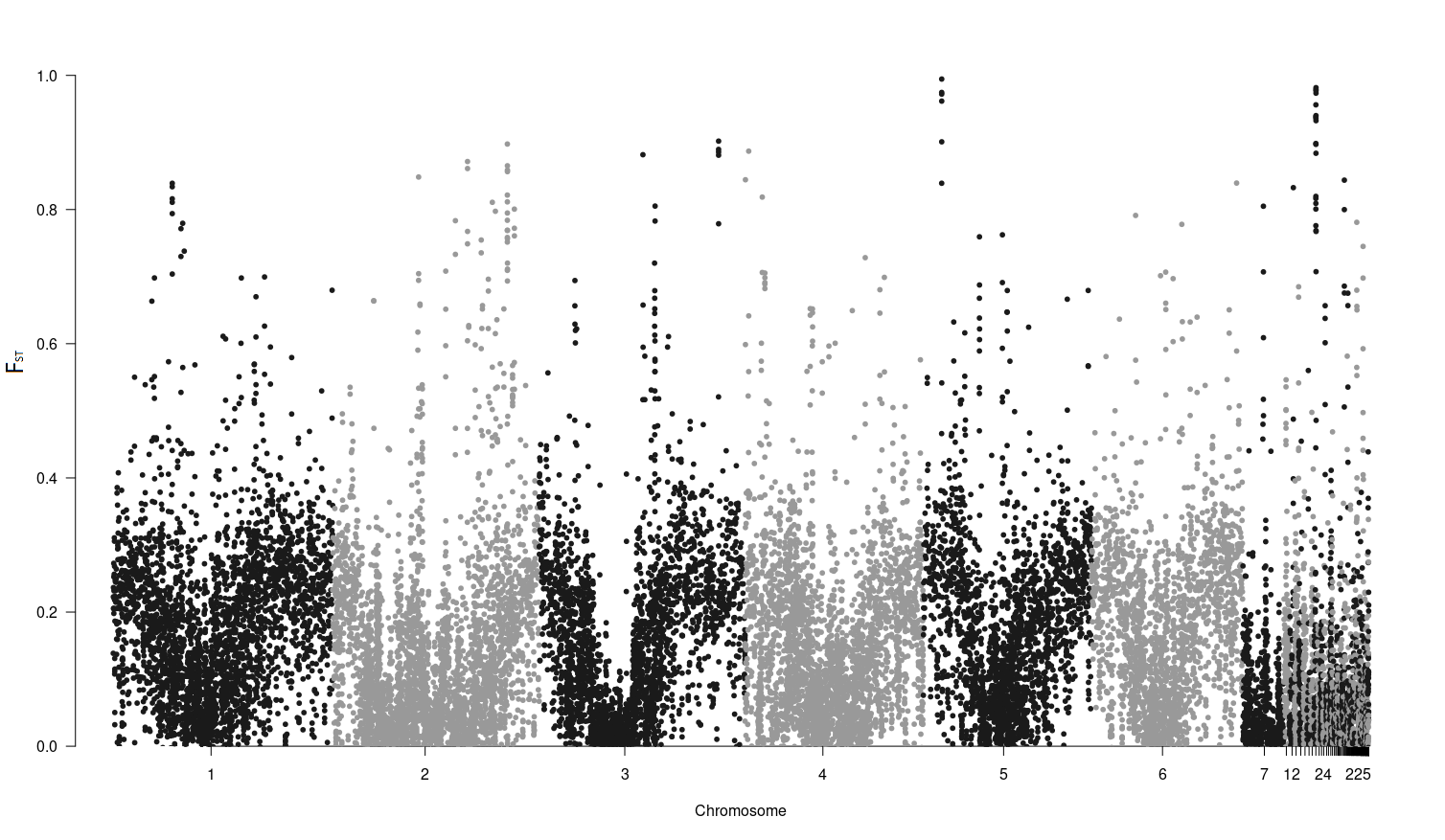


Figure S2. Distribution of SNP *F*_ST_ values from 1000 bp sliding windows across the genome (chromosomes 1 to 6 and unmapped scaffolds) calculated using all 23 sampling sites. The ‘valley’ shaped distribution of SNP *F*_ST ­­­­_values for each chromosome is due to relatively lower differentiation as SNPs approach the centromeres.


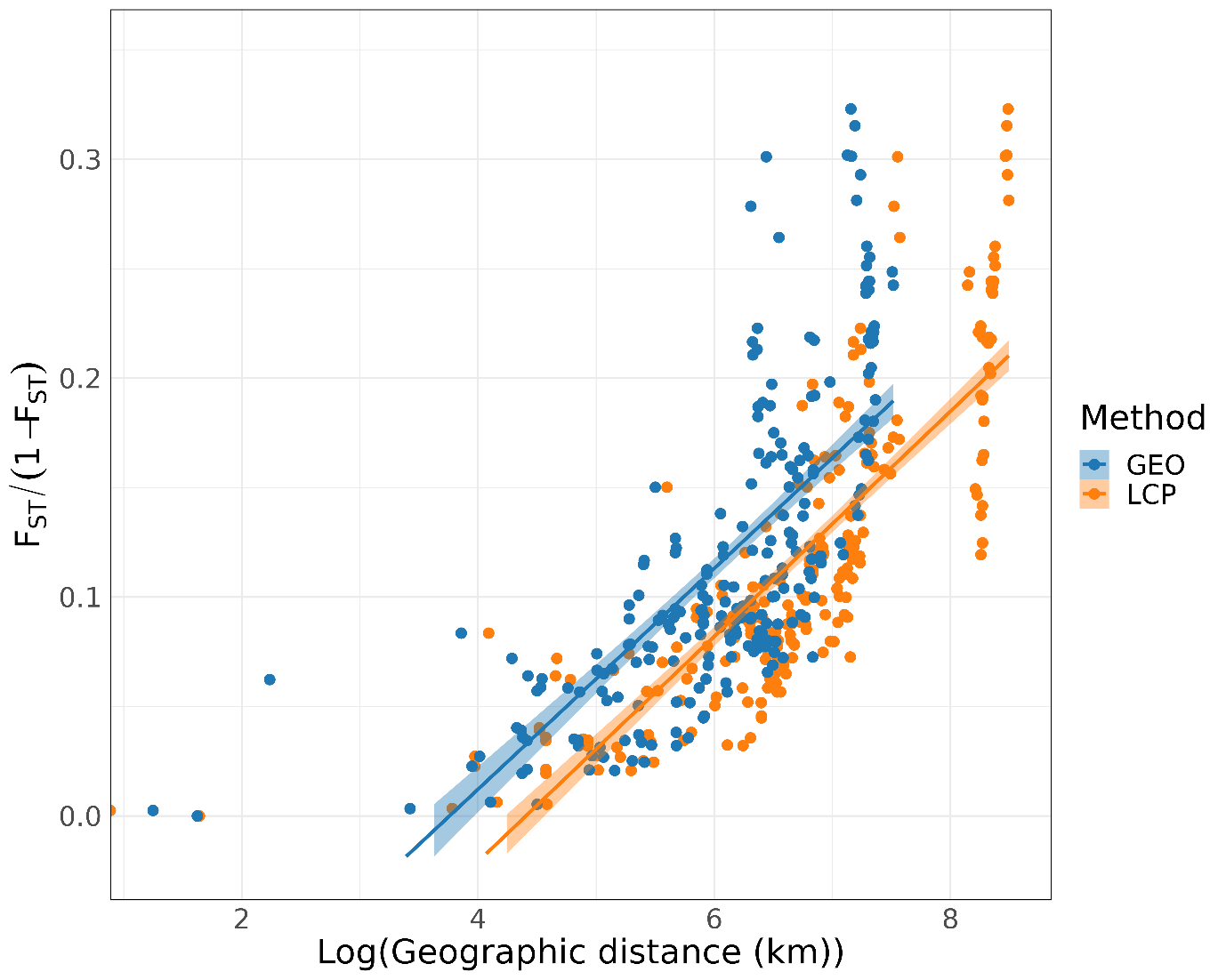


Figure S3. Linearized pairwise *F*_ST_ vs. pairwise log-transformed geographic straight-line euclidean (blue) and least-cost path following the coastline (orange) distances among sampling sites showing an isolation by distance relationship. Tsawwassen (TSW) was removed from this analysis due to its high geographic distance and *F*_ST_ value relative to all other sites.


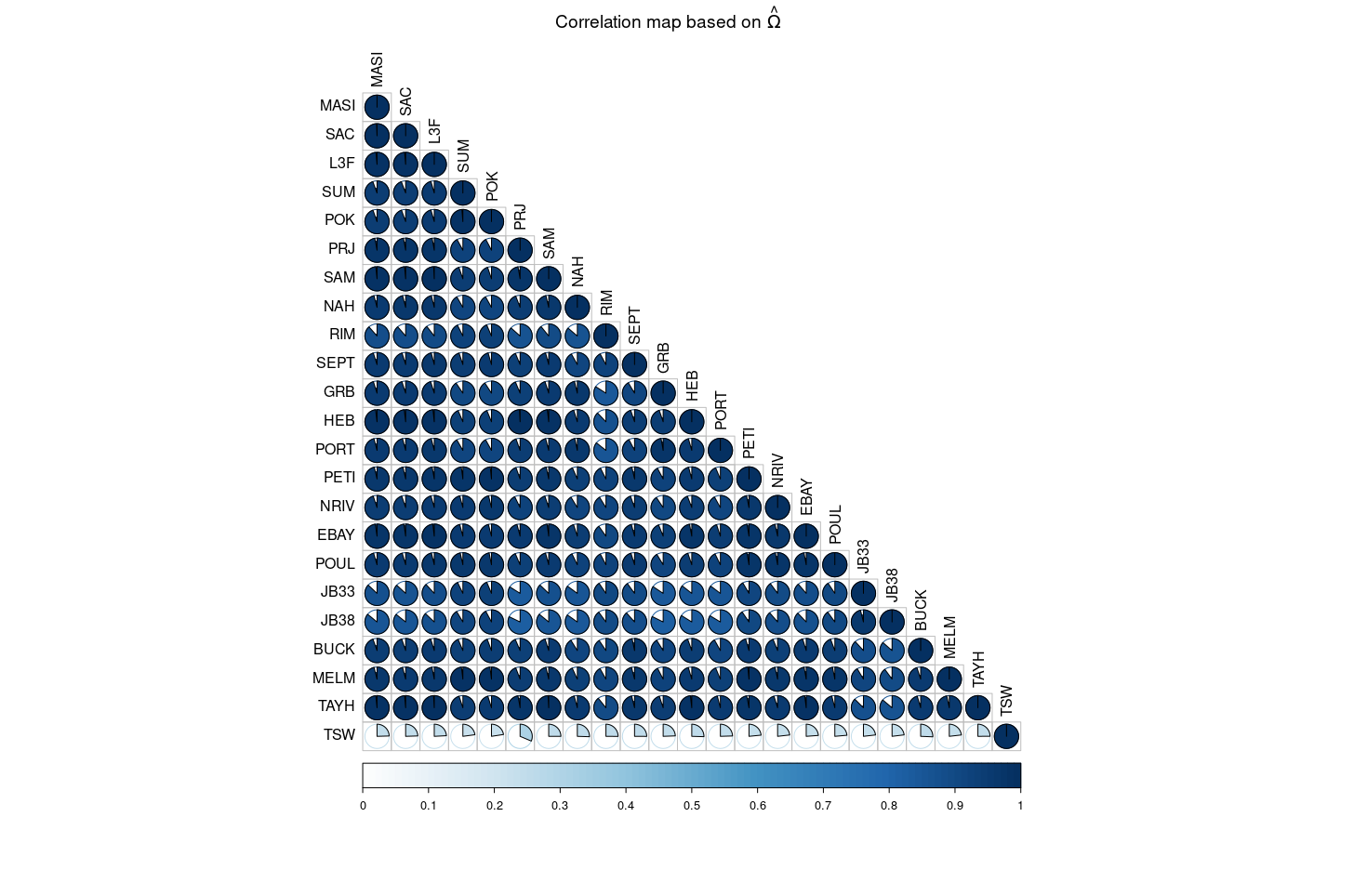


Figure S4. BayPass population covariance matrix for 23 populations used to construct the hierarchical clustering tree.


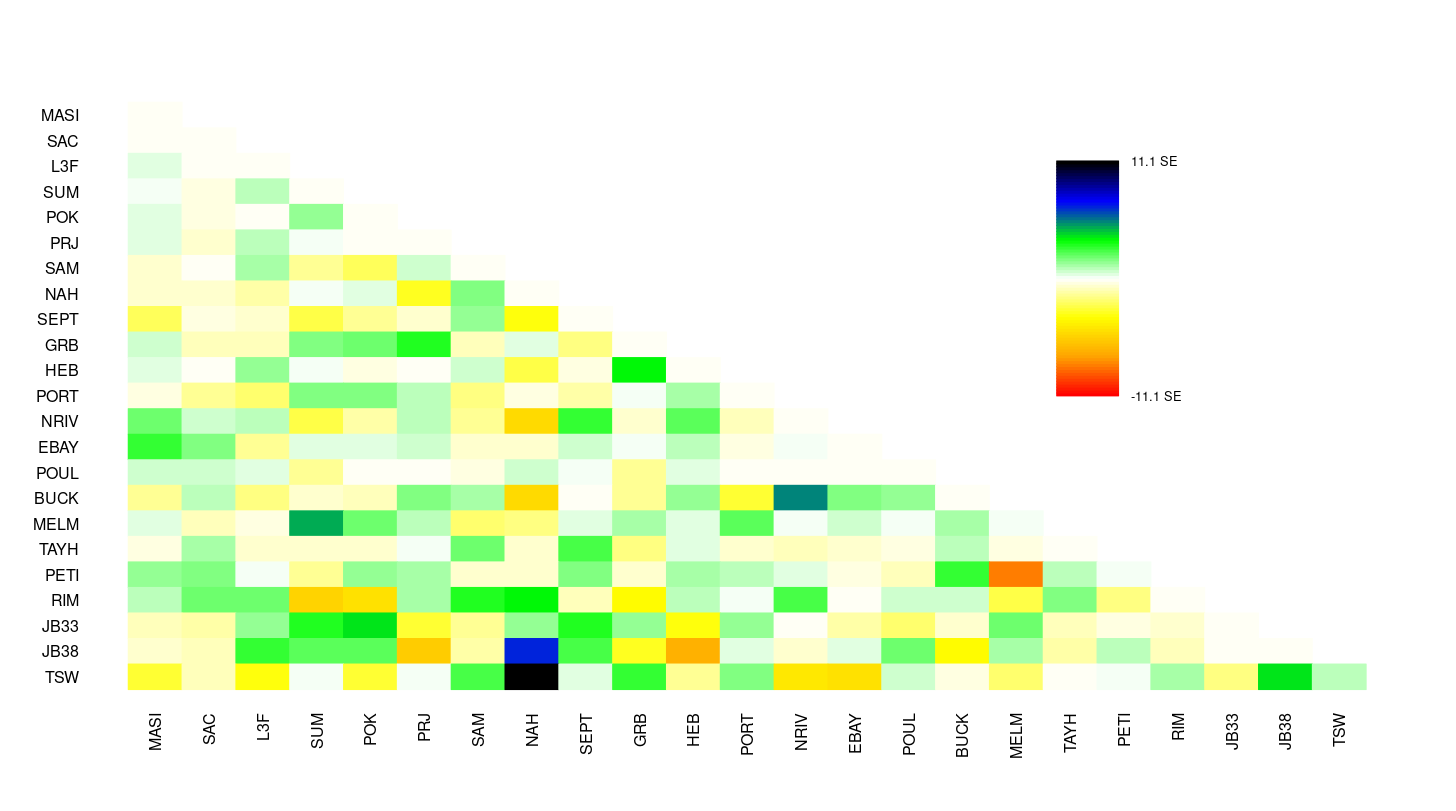


Figure S5. Distribution of residuals among 23 populations for m=6 migration events in Treemix.


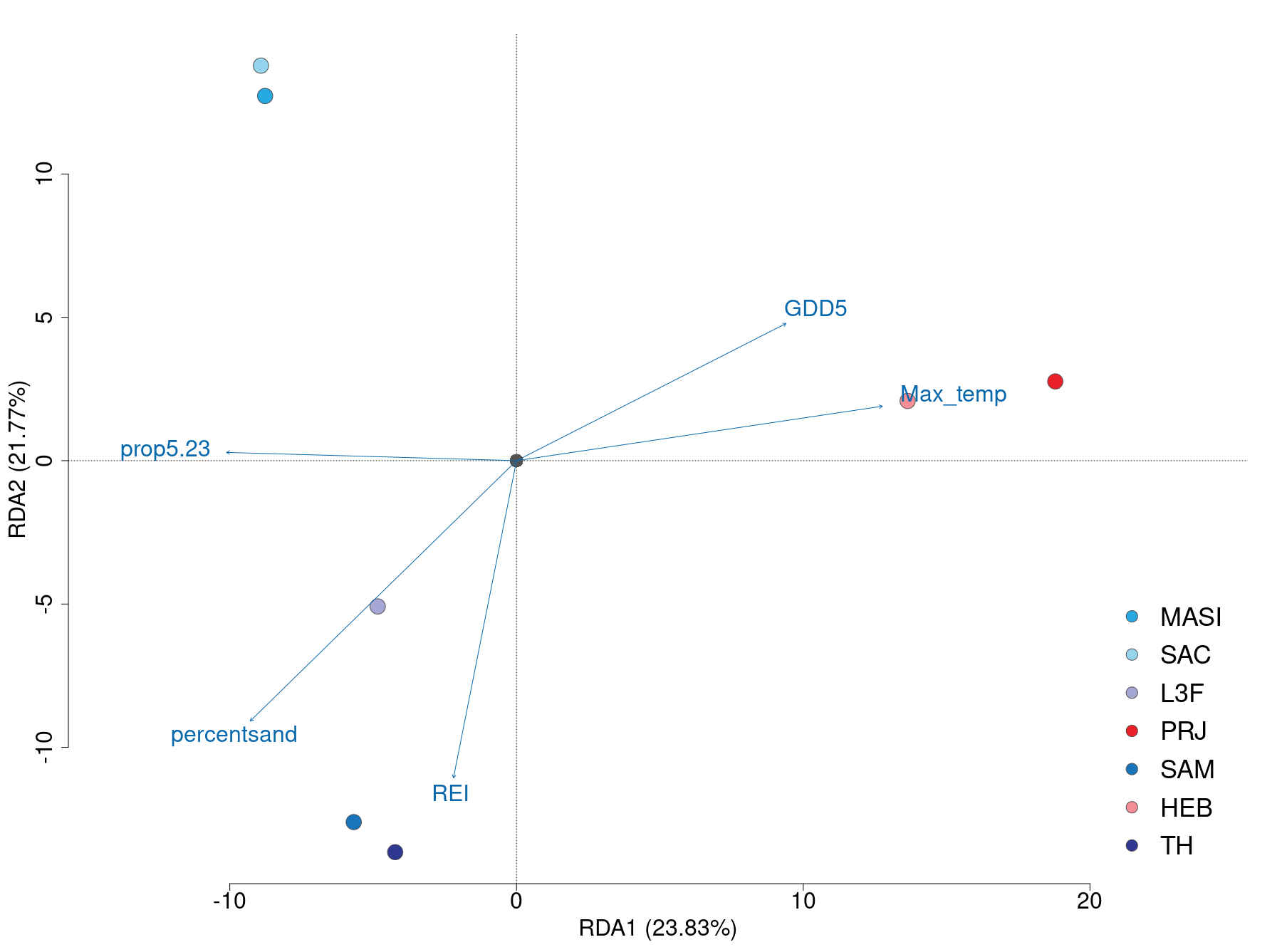


Figure S6. Redundancy analysis plots for the subset of seven mainland Nova Scotia sampling sites showing the correlations between environmental drivers and population structure on RDA axes 1 and 2. The environmental data were collected in the summer months in 2017 through 2021. REI is the relative exposure index calculated from fetch and wind data, GDD5 is the growing degree days at a baseline of 5 degrees Celsius, and prop5.23 is the proportion of days spent between 5 and 23 degrees Celsius, which represent the temperature where photosynthesis starts, and the optimal photosynthesis temperature, respectively.


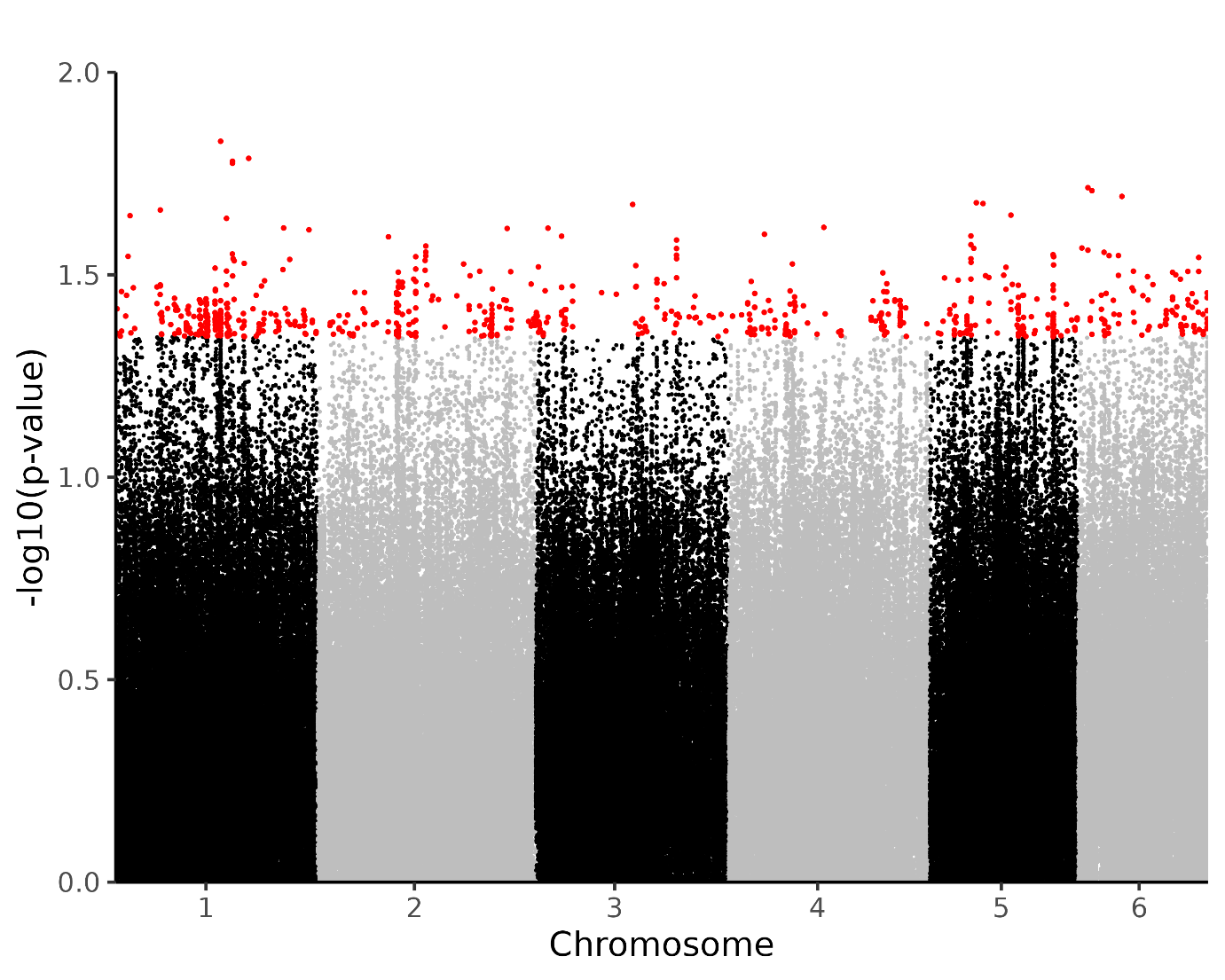


Figure S7. Distribution of environmentally-associated outlier loci (n=738) across the *Zostera marina* genome detected by redundancy analysis. These outlier loci were used to calculate the adaptive indices and genomic offset metrics for emissions scenarios RCP 4.5 and 8.5.


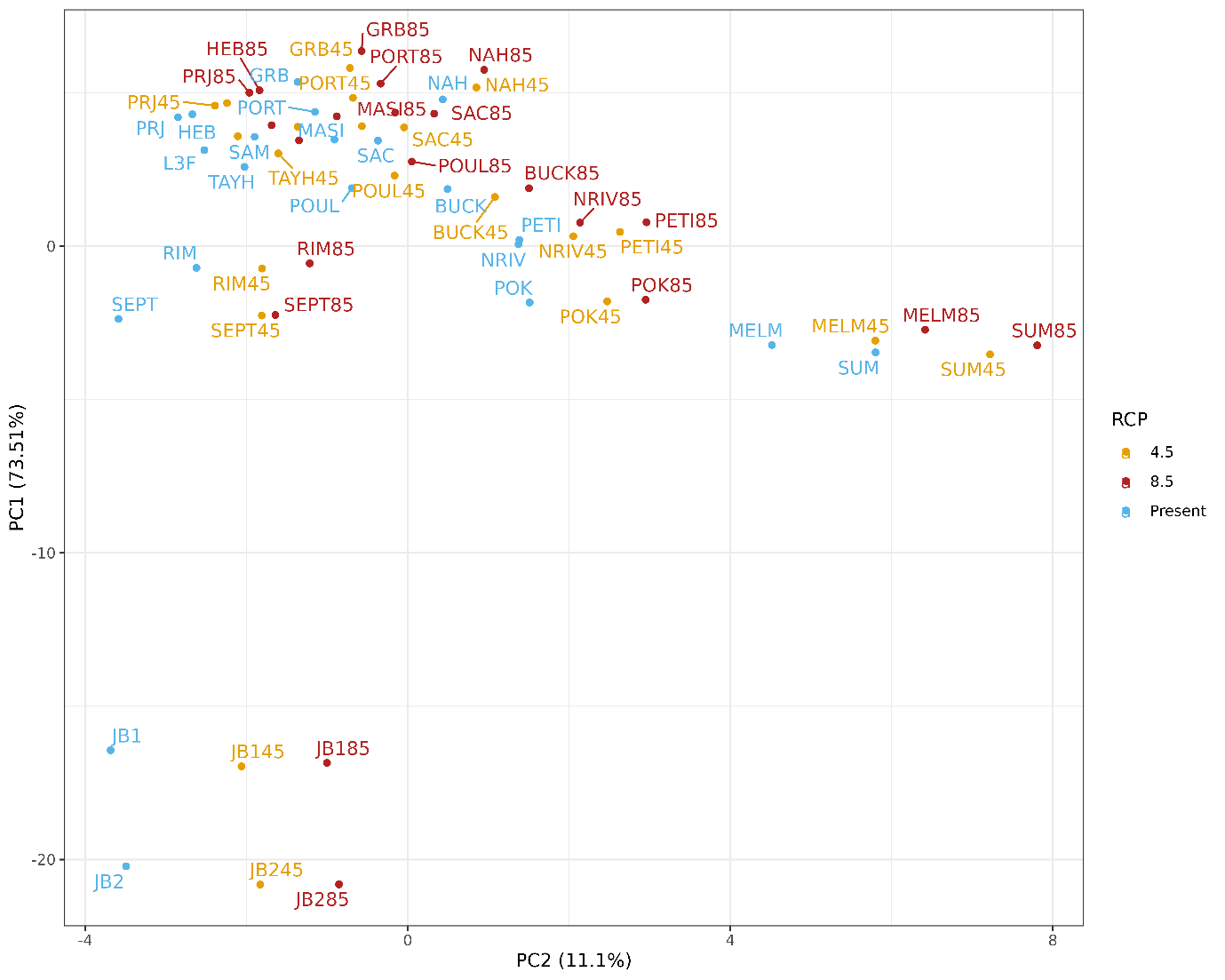


Figure S8. A principal component analysis of seasonal temperature and average salinity values for each of 21 sampling sites (excluding EBAY and TSW) shows substantial predicted climate change for east site. This is shown by shifts in each sampling location under Representative Concentration Pathway (RCP) 4.5 (yellow) and 8.5 (red) greenhouse gas emissions scenarios relative to the present day (blue text). Populations shift primarily on PC axis 2 which is associated with seasonal temperatures, whereas PC1 is primarily associated with average annual salinity.
